# Proteomic comparison of different synaptosome preparation procedures

**DOI:** 10.1007/s00726-020-02912-6

**Published:** 2020-11-19

**Authors:** Péter Gulyássy, Gina Puska, Balázs A. Györffy, Katalin Todorov-Völgyi, Gábor Juhász, László Drahos, Katalin Adrienna Kékesi

**Affiliations:** 1 MTA-TTK NAP B MS Neuroproteomics Research Group, Hungarian Academy of Sciences, Budapest, 1117 Hungary; 2 Department of Anatomy, Cell and Development Biology, Institute of Biology, ELTE Eötvös Loránd University, Budapest, 1117 Hungary; 3 Department of Ecology, University of Veterinary Medicine Budapest, Budapest, 1078 Hungary; 4 MTA-ELTE NAP Laboratory of Molecular and Systems Neurobiology, Institute of Biology, Hungarian Academy of Sciences and ELTE Eötvös Loránd University, Budapest, 1117 Hungary; 5 Laboratory of Proteomics, Institute of Biology, ELTE Eötvös Loránd University, Budapest, 1117 Hungary; 6 ELTE-NAP Neuroimmunology Research Group, Institute of Biology, ELTE Eötvös Loránd University, Budapest, 1117 Hungary; 7 MS Proteomics Research Group, Research Centre for Natural Sciences, Budapest, 1117 Hungary; 8 Department of Physiology and Neurobiology, ELTE Eötvös Loránd University, Budapest, 1117 Hungary

**Keywords:** Synapse, Synaptosome, Proteomics, Neuroproteomics, Subcellular proteomics

## Abstract

Synaptosomes are frequently used research objects in neurobiology studies focusing on synaptic transmission as they mimic several aspects of the physiological synaptic functions. They contain the whole apparatus for neurotransmission, the presynaptic nerve ending with synaptic vesicles, synaptic mitochondria and often a segment of the postsynaptic membrane along with the postsynaptic density is attached to its outer surface. As being artificial functional organelles, synaptosomes are viable for several hours, retain their activity, membrane potential, and capable to store, release, and reuptake neurotransmitters. Synaptosomes are ideal subjects for proteomic analysis. The recently available separation and protein detection techniques can cope with the reduced complexity of the organelle and enable the simultaneous qualitative and quantitative analysis of thousands of proteins shaping the structural and functional characteristics of the synapse. Synaptosomes are formed during the homogenization of nervous tissue in the isoosmotic milieu and can be isolated from the homogenate by various approaches. Each enrichment method has its own benefits and drawbacks and there is not a single method that is optimal for all research purposes. For a proper proteomic experiment, it is desirable to preserve the native synaptic structure during the isolation procedure and keep the degree of contamination from other organelles or cell types as low as possible. In this article, we examined five synaptosome isolation methods from a proteomic point of view by the means of electron microscopy, Western blot, and liquid chromatography-mass spectrometry to compare their efficiency in the isolation of synaptosomes and depletion of contaminating subcellular structures. In our study, the different isolation procedures led to a largely overlapping pool of proteins with a fairly similar distribution of presynaptic, active zone, synaptic vesicle, and postsynaptic proteins; however, discrete differences were noticeable in individual postsynaptic proteins and in the number of identified transmembrane proteins. Much pronounced variance was observed in the degree of contamination with mitochondrial and glial structures. Therefore, we suggest that in selecting the appropriate isolation method for any neuroproteomics experiment carried out on synaptosomes, the degree and sort/source of contamination should be considered as a primary aspect.

**Electronic supplementary material:**

The online version of this article (10.1007/s00726-020-02912-6) contains supplementary material, which is available to authorized users.

## Introduction

Among the nearly 16,000 canonical proteins, which are known to be expressed in a mammalian brain, thousands of proteins localize in the synapse. Despite the amount of gathered and catalogized information on synaptic proteins, it is still expected that advanced analytical methods and microscopic techniques will verify the temporary or persistent presence of novel proteins in the synapse. The synaptic transmission requires a core set of unique proteins, which is thought to be present in all synapses; however, the structural and functional diversity of synapses manifest also in their proteome. Different types of synapses, adequately to their distinct roles, contain several discrete proteins as most noticeable in the cases of receptors, ion channels, and transporters (Roy et al. 2018). Some proteins are exclusively located in the synapses, while others have multiple localizations. Proteins might differ in their expression levels, place of synthesis, trafficking route, posttranslational processing, degradation pathways, and turnover—factors that influence the proper synaptic function (Alvarez-Castelao and Schuman 2015). Not all proteins have equivalent significance in the synaptic operation, but hundreds of synaptic proteins correspond to human disease genes involved in the pathophysiology of psychiatric and neurodegenerative disorders (Bayes et al. 2011). In addition, a great portion of drugs applied in the treatment of neurological or psychiatric diseases has a primary action on synaptic proteins.

In the molecular investigation of the synaptic structures and synaptic processes, several high-throughput proteomics-based approaches are available, which enable the analysis of a particular portion of the synaptic proteome. Techniques based on immunoaffinity and affinity purification using highly specific antibodies, chemical ligands, aptamers or combinatorial peptide libraries are able to selectively isolate synaptic target proteins or complexes of target proteins. Isolation of the target protein or its complex from the background matrix gives scope for the analysis of the structural properties and/or interaction partners of the given protein (Brinkmalm et al. 2014). For instance, by the isolation of kinesin molecular motor proteins (like Kif5C and Kif3A) and their cargo proteins, it is feasible to examine the portion of the synaptic proteome, which is synthetised in the cell body and transported to distal neuronal processes, such as dendrites, axons and synapses (Liu et al. 2014). For a more comprehensive, system-wide understanding of synaptic structure and function, synaptosomes serve as distinguished research objects.

Unlike cellular organelles, synaptosomes are artificial objects with an average diameter of 0.5–1 µm. They are formed when the neuronal tissue is homogenized in an isotonic milieu and the nerve endings torn apart from their axons and surrounding glia and the lipid bilayer reseals. As a result of the mechanical damage, some of the nerve endings become damaged due to their size or shape and lose their internal content before the membrane closes up, but the majority of synaptosomes preserves the whole apparatus for synaptic transmission. Synaptosomes contain the presynaptic nerve ending with the synaptic vesicles, synaptic mitochondria, and in most cases, a part of the postsynaptic membrane and the postsynaptic density attached to the synaptosome surface. Since 1964, when the group of Whittaker first isolated, synaptosomes became essential objects of synaptic transmission-related research closely mimicking the functions of nerve terminals in vivo (Whittaker et al. 1964). During a proper isolation process, the synaptosomes retain their pre- and postsynaptic morphological and functional characteristics, membrane potential and show high activity for several hours. They remain able to store, release, and take up neurotransmitters. In addition, synaptosomes can be isolated from post mortem human nervous tissue subserving the molecular analysis of synaptopathies underlying human neurological and psychiatric disorders, and are starting points for further subcellular fractionation processes for the isolation of synaptic vesicles, presynaptic detergent soluble membrane fraction or the postsynaptic density (Dunkley et al. 2008; Tenreiro et al. 2017).

Proteomics experiments using synaptosomes has greatly contributed to the understanding of the molecular architecture and physiological or pathological processes of synapses (Dieterich and Kreutz 2016). Synaptosomes has been isolated from peripheral or central nervous tissues from various species and human biopsies or postmortem samples (DeGiorgis et al. 2005; Singh et al. 2009; Jhou and Tai 2017). Microdissected tissues were used for the molecular characterization of synapses from distinct anatomical regions while fluorescent-activated sorting enabled the isolation and proteomic characterization of subpopulations of synapses based on their neurotransmitter system (Biesemann et al. 2014). High-resolution separation techniques coupled on or offline to sensitive mass spectrometers are capable of the identification and absolute or relative quantification of thousands of proteins and/or posttranslational modifications in a single experiment (Andrade et al. 2007; Craft et al. 2013; Ren et al. 2014). Cross-linking approaches or native electrophoretic separation are widely applied to reveal the protein composition of macromolecular complexes of synaptic transmission machineries (Holman et al. 2007; Gonzalez-Lozano et al. 2020).

Performing neuroproteomic analysis on synaptosome samples has several benefits. (1) Although state-of-the-art analytical techniques permit the simultaneous qualitative and quantitative analysis of thousands of proteins, none of the available separation and protein identification methods can sufficiently resolve the proteins/protein isoforms present in the heterogeneous nervous tissue comprising multiple cell types. By isolating synaptosomes, the sample can be depleted from the less relevant but highly abundant proteins from other organelles and the reduced complexity opens door to the analysis of less abundant but functionally important proteins. (2) In the central nervous system, the heterogeneous population of glial cells makes up around half of the cells, therefore around half of the protein content in a whole tissue homogenate is of glial origin (Bartheld et al. 2016). Since there is a substantial overlap in the protein expression profile of glial cells and neurons, in a case of a ubiquitous protein present in both glial and neuronal cells, its detected structural or quantitative alteration under the experimental condition cannot be attributed to a given cell type. Ideally, synaptosome preparations lack glial contamination rendering all proteins present in the sample certainty of their neuronal origin. (3) Synaptosome fractions solely contain proteins which have a relevant role in shaping the structure and function of the synapse. Among proteins with multiple localization, only the synapse-related isoform is present in the sample, like among mitochondria, only the idiosyncratic synaptic mitochondria, having peculiar functions and proteome profile, is included.

After the homogenization and synaptosome formation, the synaptosome fraction should be enriched from the whole tissue homogenate. Several methods, each having advantages and drawbacks, exist for the isolation of synaptosomes, although, there is not a single procedure, which is optimal for every research purpose. The method of choice should depend on the purpose of the experiment and on the applied analytical methods. The quality of the synaptosome sample highly depends on the isolation procedure, and in practice, the sample inevitably contains contamination to some extent. Synaptosomes can be investigated for different purposes, by several research approaches, for which distinct isolation processes are compatible or optimal. For some purposes (e.g., specific enzyme assays) the only goal is to enrich the synaptic components to improve the sensitivity of the analysis, whilst other contaminating structures do not distort the experimental outcome. In other experiments (e.g., neurotransmitter release/reuptake analysis), the main objectives are to preserve the structural and functional integrity (viability) as long as possible. In proteomic experiments, it is substantial to preserve the proper molecular structure of the synaptosome during the isolation process, to extract sufficient sample material to downstream analysis, and to minimize the degree of contamination from other cell types or organelles.

In our recent work, we compared five different synaptosome isolation methods from a proteomic point of view. We analyzed samples obtained by three density gradient centrifugation methods, by one membrane filtration process, and using a commercially available kit, by the means of electron microscopy, Western blot, and liquid chromatography-tandem mass spectrometry to characterize their ability to preserve synaptic structures, their degree of contamination, and their proteome.

## Materials and methods

### Animals

Adult male Wistar rats (4 months old, weighing 350–400 g, purchased from Toxi-Coop Ltd., Budapest, Hungary) were used in all experiments. Animals were housed under standard laboratory conditions in a 12 h light–dark cycle (light was on from 09:00 AM to 09:00 PM) in air-conditioned rooms at 22 ± 2 °C. Food and water were supplied ad libitum. Handling and experimentation on animals were performed in accordance with The Code of Ethics of the World Medical Association (Declaration of Helsinki), the Council Directive 86/609/EEC, the Hungarian Act of Animal Care and Experimentation (1998, XXVIII), and local regulations for the care and use of animals for research. All efforts were taken to minimize the animals’ pain and suffering and to reduce the number of animals used.

### Synaptosome preparation

Rats were deeply anesthetized with i.p. urethane administration and decapitated, the brains were quickly removed and washed in dry ice-cooled artificial cerebrospinal fluid (ACSF). Cerebral cortices were dissected on a dry ice-cooled plate. To minimize the heterogeneity of the samples arise from individual biological and anatomical differences, the cortices from different animals were sliced into small pieces with a pre-cooled scalpel on a dry-ice-cooled plate and mixed. Portions of the resulting pool were used in all synaptosome preparation methods. 150 mg of cortical tissue was used in each experiment and all further steps were performed either on ice or in a cold room to avoid post mortem degradation. Five different methods were performed.

#### #1 Method

Synaptosome enrichment was performed following the protocol of Phillips et al*.* (2001) and Hahn et al. (2009) with minor modifications. Briefly, 150 mg cortical tissue was homogenized in 1 ml of homogenization buffer containing 320 mM sucrose, 0.1 mM CaCl_2_, 1 mM MgCl_2_, supplemented with Protease and Phosphatase Inhibitor Cocktails (Sigma-Aldrich, St. Louis, MO, USA) with 40 strokes in a Dounce type glass homogenizer (Kontes Glass Co., Vineland, NJ, USA) using the small clearance pestle. The homogenate was adjusted to 1.25 M sucrose, 0.1 mM CaCl_2_ to a total volume of 5 ml. The sample was transferred to a centrifuge tube, 5 ml of 1 M sucrose solution was overlaid on it, and was centrifuged at 100,000×*g*_(max)_ for 3 h in an SW-40 swinging-bucket rotor. The synaptosome fraction was collected as a band at the interface. The sample was diluted with 5 × volumes of 0.1 mM CaCl_2_ and centrifuged at 15,000×*g* for 20 min and the pellet was precipitated via incubation with ice-cold acetone at − 20 °C overnight. The next day, the sample was spun down, the acetone was removed and the pellet was allowed to dry.

#### #2 Method

Synaptosome enrichment was achieved following the method published by Witzman et al. with minor modifications (Witzmann et al. 2005). In brief, 150 mg tissue was homogenized in 1 ml of homogenization buffer containing 320 mM sucrose, 20 mM HEPES–KOH pH 7.4, supplemented with Protease and Phosphatase Inhibitor Cocktails (Sigma-Aldrich, St. Louis, MO, USA) with 40 strokes in a Dounce type glass homogenizer (Kontes Glass Co., Vineland, NJ, USA) using the small clearance pestle. One ml of homogenization buffer was added and the homogenate was centrifuged at 1000×*g* for 5 min at 4 °C. The supernatant was further centrifuged at 17,000×*g* for 15 min at 4 °C and the resultant pellet was resuspended in 1 ml of homogenization buffer. The sample was pipetted on a 10 ml gradient built up by 5 ml of 0.8 M sucrose, 20 mM HEPES–KOH pH 7.4 layered on the top of 5 ml 1.2 M sucrose, 20 mM HEPES–KOH pH 7.4 and centrifuged at 54,000×*g*_(av)_ for 90 min at 4 °C in an SW-40 swinging-bucket rotor. The band at the interface was collected with a needle as the synaptosome fraction, diluted to 9 ml with homogenization buffer, and centrifuged at 20,000×*g* for 30 min at 4 °C. The resultant pellet was precipitated with ice-cold acetone overnight. The next day, the sample was spun down, the acetone was removed and the pellet was allowed to dry.

#### #3 Method

Synaptosomes were isolated according to the protocol of Tandon et al. (1998) with minor modifications. Briefly, the 150 mg of cortical tissue was homogenized in 1 ml of homogenization buffer containing 320 mM sucrose, 5 mM HEPES–KOH pH 7.4, supplemented with Protease and Phosphatase Inhibitor Cocktails (Sigma-Aldrich, St. Louis, MO, USA) with 40 strokes in a Dounce type glass homogenizer (Kontes Glass Co., Vineland, NJ, USA) using the small clearance pestle. One ml of homogenization buffer was added and the homogenate was centrifuged at 1000×*g* for 15 min at 4 °C. The supernatant was further centrifuged at 13,300×*g* for 15 min at 4 °C and the resultant pellet was resuspended in 2.5 ml of homogenization buffer. The sample was overlaid on the top of a three-step gradient of 4 ml of 13% (wt/vol) Ficoll, 1 ml of 9% (wt/vol) Ficoll, 4 ml of 5% (wt/vol) Ficoll dissolved in homogenization buffer and layered on each other and centrifuged at 86,000×*g*_(max)_ for 35 min at 4 °C in an SW-40 swinging-bucket rotor. The synaptosome fraction was collected with a needle from the interface between the 13 and 9% Ficoll layers. The fraction was diluted to 9 ml with homogenization buffer and centrifuged at 20,000×*g* for 30 min at 4 °C and the resultant pellet was precipitated with ice-cold acetone at − 20 °C overnight. Subsequently, the sample was spinned down, the acetone was removed and the pellet was allowed to dry.

#### #4 Method

The synaptosome preparation was conducted based on the protocol of Bajor et al. (2012) with minor modifications. In brief, 150 mg of cortical tissue sample was homogenized in 1 ml of homogenization buffer containing 320 mM sucrose, 5 mM HEPES–KOH pH 7.4, supplemented with Protease and Phosphatase Inhibitor Cocktails (Sigma-Aldrich, St. Louis, MO, USA) with 40 strokes in a Dounce type glass homogenizer (Kontes Glass Co., Vineland, NJ, USA.) using the small clearance pestle. After the homogenization, 1 ml of homogenization buffer was added and centrifuged at 1000×*g* for 10 min at 4 °C. The supernatant was loaded into a 2 ml syringe and gravity filtered through a 5 µm pore size hydrophilic membrane filter (Merck Millipore, Billerica, MA, USA) held in a 13 mm diameter filter holder, and later the filtrate was centrifuged at 13,200×*g* for 30 min at 4 °C. The pellet was precipitated with ice-cold acetone overnight. The next day, the sample was spun down, the acetone was removed and the pellet was allowed to dry.

#### #5 Method

The synaptosome enrichment was performed using the Syn-PER™ Synaptic Protein Extraction Reagent (Thermo Fisher Scientific, Waltham, MA, USA) following the manufacturer’s recommendations. Briefly, 150 mg of cortical tissue was homogenized in 1.5 ml of Syn-PER reagent supplemented with Protease and Phosphatase Inhibitor Cocktails (Sigma-Aldrich, St. Louis, MO, USA) with 40 strokes in a Dounce type glass homogenizer (Kontes Glass Co., Vineland, NJ, USA) using the small clearance pestle. The homogenate was centrifuged at 1200 × *g* for 10 min at 4 °C, the pellet was discarded and the supernatant was further centrifuged at 15,000×*g* for 20 min at 4 °C. The pellet was precipitated with ice-cold acetone overnight. The next day, the sample was spun down, the acetone was removed and the pellet was allowed to dry.

### Preparation of whole cerebral cortex homogenate

The brain tissue was mechanically homogenized in lysis buffer (7 M urea, 2 M thiourea, 4% (wt/vol) CHAPS, 20 mM Tris, 5 mM MgCl_2_, 50 mM DTT) using an IKA-Turrex blender (IKA-Werke, Staufen, Germany) for 10 × 10 s at 15,000 rpm on ice. The homogenate was later sonicated for 10 × 10 s (Ultrasonic Processor, Cole Palmer, Niles, IL, USA) on ice. The protein content of the pellet was precipitated with ice-cold acetone overnight. The next day, the sample was spun down, the acetone was removed, and the pellet was allowed to dry.

### Western blot analysis of synaptosome enrichment

Acetone-precipitated proteins from the synaptosomes prepared with the five different methods and the protein content of the whole cerebral cortex homogenate were solubilized in lysis buffer (7 M urea, 2 M thiourea, 4% (wt/vol) CHAPS, 20 mM Tris, 5 mM MgCl_2,_ 50 mM DTT) and sonicated on ice until completely dissolved. The protein concentration was determined using the 2-D Quant Kit (GE Healthcare, Little Chalfont, UK) according to the manufacturer’s recommendations and samples containing 20 µg protein amount were mixed with two-fold concentrated sample buffer (8% (wt/vol) SDS, 3% (wt/vol) DTT, 24% (vol/vol) glycerol, 0.2% (wt/vol) bromophenol blue, 100 mM Tris–HCl (pH 6.8)) to a total volume of 20 µl and were boiled at 96 °C for 5 min. Proteins were separated on a discontinuous 10% (wt/vol) polyacrylamide gel by Tricine-SDS electrophoresis and transferred onto a Hybond-LFP PVDF membrane (GE Healthcare, Little Chalfont, UK). The blots were blocked with 5% (wt/vol) bovine serum albumin (BSA) in Tris-buffered saline with 0.05% Tween-20 (TBS-T) for 1 h. Subsequently, the membranes were incubated overnight in the blocking buffer with the following primary antibodies: anti-Idh3a antibody (1:500 dilution; Proteintech, Rosemont, IL, USA; Cat.no.: 15909-1-AP), anti-Gfap antibody (1:100 dilution; Agilent Technologies (Dako), Santa Clara, CA, USA; Cat.no.: Z0334), anti-Psd-95 antibody (1:2000 dilution; Thermo Fisher Scientific; Cat.no.: MA1-046), anti-Vdac1 antibody (1:2500 dilution; Merck Millipore, Billerica, MA, USA; Cat.no.: AB10527), anti-Synaptophysin antibody (1:1000 dilution; Abcam, Cambridge, UK; Cat.no.: ab8049) and anti-Mbp antibody (1:100 dilution; Agilent Technologies (Dako), Santa Clara, CA; Cat.no.: A0623). Anti-Actin antibody (1:1,000 dilution; Abcam, Cambridge, UK; Cat.no.: ab1801) was used to detect levels of actin as a loading control. Next day, the membranes were washed 4 × 5 min with TBS-T and were incubated with Cy3-conjugated AffiniPure Donkey Anti-Rabbit IgG (H + L) (1:1000 dilution; Jackson ImmunoResearch Laboratories, West Grove, PA, USA; Cat.no.: 711-165-152 for Idh3a) or Alexa Fluor 647-conjugated AffiniPure Donkey Anti-Mouse IgG (H + L) (1:1000 dilution; Jackson ImmunoResearch Laboratories; Cat.no.: 715-605-151 for synaptophysin and Psd-95) or Alexa Fluor 647-conjugated Donkey Anti-Rabbit IgG (H + L) (1:1000 dilution, Jackson ImmunoResearch Laboratories; Cat.no.: 711-605-152 for Mbp) or Alexa Fluor 488-conjugated AffiniPure Donkey Anti-Rabbit IgG (1:1000 dilution; Jackson ImmunoResearch Laboratories; Cat.no.: 711-545-152 for Gfap, Vdac1, and actin). After washing steps with TBS-T and then with TBS, the bands were visualized using a Typhoon TRIO + fluorescent laser scanner (GE Healthcare, Little Chalfont, UK). Densitometry analysis of fluorescence intensities was performed with the ImageJ program (https://imagej.nih.gov/ij/, National Institutes of Health, Bethesda, MD, USA). Densitometry data were normalized to the loading control.

### Electron microscopic analysis of synaptosome enrichment

#### Sample processing and electron microscopy

Synaptosome fractions were fixed with the mixture of 2% (wt/vol) formaldehyde (freshly depolymerized from paraformaldehyde) and 1% (wt/vol) glutaraldehyde in 0.1 M sodium cacodylate buffer (pH 7.4) for 1 h at room temperature. After rinsing thoroughly in 0.05 M Tris buffer the samples were post-fixed with the solution of 0.5% (wt/vol) osmium tetroxide and 0.75% (wt/vol) potassium hexacyanoferrate for 1 h. Then, samples were stained with 2% (vol/vol) aqueous uranyl acetate for 30 min and were dehydrated via a graded series of ethanol. Fixed and stained synaptosome samples were embedded in LR white resin (Sigma-Aldrich) according to the manufacturer’s instructions and ultrathin sections (60–70 nm) were produced and collected onto 300 mesh copper grids. Samples were examined with a JEM-1011 electron microscope (JEOL, Tokyo, Japan) operating at 60 kV. Images were taken with an 11 megapixel Olympus Morada camera.

#### Morphometric analysis

Morphometric analysis of images was carried out to quantify the purity of the different sample preparations and thereby determining the efficacy of the five purification procedures. Two grids were used for each sample and 6 images per grids were taken at 25,000× magnification achieving systematic, uniform and random sampling. The areas of the structures were measured in a 6 µm × 4 µm frame of the 12 images per preparations by using the ImageJ software. Objects of the samples were manually clustered into one of the groups as follows: certainly synaptosomes (objects with well-defined structures containing synaptic vesicles or postsynaptic density), most likely synaptosomes (rounded objects with empty re-sealed membrane without synaptic vesicles or postsynaptic density but with similar size range and shape like synaptosomes) and non-synaptosomal structures (mostly extrasynaptosomal mitochondria and other different sized unidentifiable structures). Additional micrographs at lower magnification (10,000×) were acquired in the case of the #5 Method (Syn-PER) samples since this preparation contained large-sized non-synaptosomal structures which have often extended outside of the examination frames, and statistical analyses were corrected accordingly.

For evaluation of area density values, we manually cut out the areas covered by synaptosomal, most likely synaptosomal, and non-synaptosomal structures on the images, summed their sizes separately, and calculated the amount of the three distinct clusters (overall cluster area size in % of the total area covered). In addition, we prepared frequency distribution histograms on the area size distribution of certainly synaptosomal structures in all samples to determine whether the different purification procedures resulted in the fragmentation of the synaptosomal compartments or they remained intact.

#### Statistical analysis

Statistical analyses were performed using the statistical package IBM SPSS Statistics Version 17. Kruskal–Wallis (K–W) and post hoc Mann–Whitney (M–W) tests were applied to determine if there were differences in the cluster area sizes of the synaptosome preparations between the different purification procedures. In addition, we analysed the differences between the distinct sub-synaptosomal groups pooled from the same purified fraction.

### Enzymatic digestion and sample preparation for mass spectrometry

The precipitated synaptosome samples were resuspended in lysis buffer (7 M urea, 2 M thiourea, 20 mM Tris, 5 mM Mg(Ac)_2_, 50 mM DTT) and were sonicated on ice until completely dissolved. The protein concentration was determined using the 2-D Quant Kit (GE Healthcare, Little Chalfont, UK) and the proteins were digested following the filter-aided sample preparation method published by Wisniewski et al. with minor modifications (Wisniewski et al. 2009). Briefly, 150 µg of the samples were diluted with urea buffer (8 M urea, 100 mM Tris–HCl pH 8.5) to a total volume of 200 µl, transferred to a Microcon YM-30 filter device (Merck Millipore) and centrifugated at 14,000×*g* for 15 min at room temperature. Then, 200 µl urea buffer was added to the samples and spun down again. For protein carbamidomethylation, 100 µl of IAA solution (50 mM iodoacetamide, 8 M urea, 100 mM Tris–HCl pH 8.5) was pipetted onto the filter and mixed at 450 rpm at room temperature for 3 min in a thermo-mixer. The samples were incubated for 45 min at room temperature in the dark without mixing and were centrifugated for 10 min. One hundred µl of urea solution was added to the samples and spun down for 15 min, and this step was repeated twice. Subsequently, 100 µl of 50 mM NH_4_HCO_3_ was added and the samples were centrifugated for 10 min and this step was repeated twice. The proteins were recovered from the filter by a reverse spin at 1500×*g* for 3 min and 100 µl of digestion solution (0.1% (wt/vol) RapiGest (Waters, Milford, MA, USA), 50 mM NH_4_HCO_3_) and trypsin (Sequencing grade, modified, Promega, Madison, WI, USA) in a 1:50 ratio was added. The samples were digested overnight at 37 °C. The next day, the reaction was terminated by adding 4 µl of formic acid (FA) and the samples were desalted on a Pierce C-18 spin column (Thermo Scientific, Sunnyvale, CA, USA) following the manufacturer’s instructions and dried in a speed-vac.

### Liquid chromatography-mass spectrometry analysis

The LC–MS/MS-based protein identification of the proteins present in each synaptosome sample was performed using a Maxis II ETD QqTOF (Bruker Daltonics, Bremen, Germany) coupled to an Ultimate 3000 nanoRSLC system (Dionex, Sunnyvale, CA, USA) under the control of Hystar v.3.2 (Bruker Daltonics, Bremen, Germany). The air-dried, digested synaptosome samples were dissolved in 30 µl of 2% (vol/vol) acetonitrile (AcN), 0.1% (vol/vol) FA out of which 1 µl were injected onto an Acclaim PepMap100 C-18 trap column (100 µm × 20 mm, Thermo Scientific, Sunnyvale, CA, USA). Sample preconcentration and desalting were performed with 0.1% (vol/vol) TFA for 8 min with a flow rate of 5 µl/min. The tryptic peptides were separated on an ACQUITY UPLC M-Class Peptide BEH C18 column (130 Å, 1.7 µm, 75 µm × 250 mm, Waters, Milford, MA, USA) at 48 °C using a flow rate of 300 nl/min. The eluent A was 0.1% (vol/vol) FA and the eluent B was AcN, 0.1% (vol/vol) FA. The gradient started with 4% B from 0 to 11 min, followed by a 120 min gradient to 50% B, and then, the concentration of the solvent B was elevated to 90% in 1 min and kept there for 10 min. Sample ionization was achieved in positive electrospray ionization mode via a CaptiveSpray nanoBooster ion source. The nanoBooster pressure was 0.2 bar, the capillary voltage was set to 1,300 V, the drying gas was heated to 150 °C and the flow rate was 3 l/min. External mass calibration was performed via direct infusion using a low concentration tuning mix (Agilent Technologies, Santa Clara, CA, USA) and internal mass calibration was performed for each run using sodium formate via lock mass. The ion transfer parameters were set as follows: prepulse storage 10 µs, collision transfer 10 µs, quadrupole ion energy 5 eV, Funnel 1 RF 400 Vpp, Multipole RF 400 Vpp. The collision RF was set to 1200 Vpp and the ion transfer time was 120 µs. The MS spectra were recorded with a fixed cycle time of 2.5 s over the mass ranges of *m/z* 300–650, 650–850, 850–2200 and 150–2200 in four consecutive runs at 3 Hz with a minimal precursor mass of 322 *m/z*. The CID was performed at 16 Hz for abundant precursors and 4 Hz for ones of low abundance. For fragmentation, only multiply charged peptides were chosen, while singly charged peptides were excluded from the analysis. The collision energy for precursor signals was set automatically according to the manufacturer’s recommendations, based on the isolation *m/z*, isolation mass range width, and charge state of the ions. An active exclusion of 2 min after 1 spectrum was used, except if the intensity of the precursor was elevated threefold. For protein content analysis, raw data were recalibrated using the Compass DataAnalysis software 4.3 (Bruker Daltonics). The raw data from the consecutive runs were merged and the samples were matched with the *Rattus norvegicus* SwissProt database using the Mascot server v.2.5 (Matrix Science, London, UK). The parameters for the Mascot search were set as follows: trypsin as the enzyme, max. 2 missed cleavages were allowed and cysteine carbamidomethylation as fixed and methionine oxidation as variable modification were searched. Precursor tolerance and MS/MS tolerance were set to 7 ppm and 0.05 Da, respectively. Decoy database was generated by Mascot and the false discovery rate was set at less than 1% in every search result. Proteins with a minimum of two identified unique peptides were accepted.

## Results and discussion

### Morphometric analysis of synaptosome preparations

The distributions of synaptosomal and non-synaptosomal structures were examined in the different synaptosomal preparations. Three groups were generated of the structures present in the five synaptosomal samples following distinct purification procedures (#1-#5 Method): certainly synaptosomal structures, most likely synaptosomal structures, and non-synaptosomal structures.

**Fig. 1:**
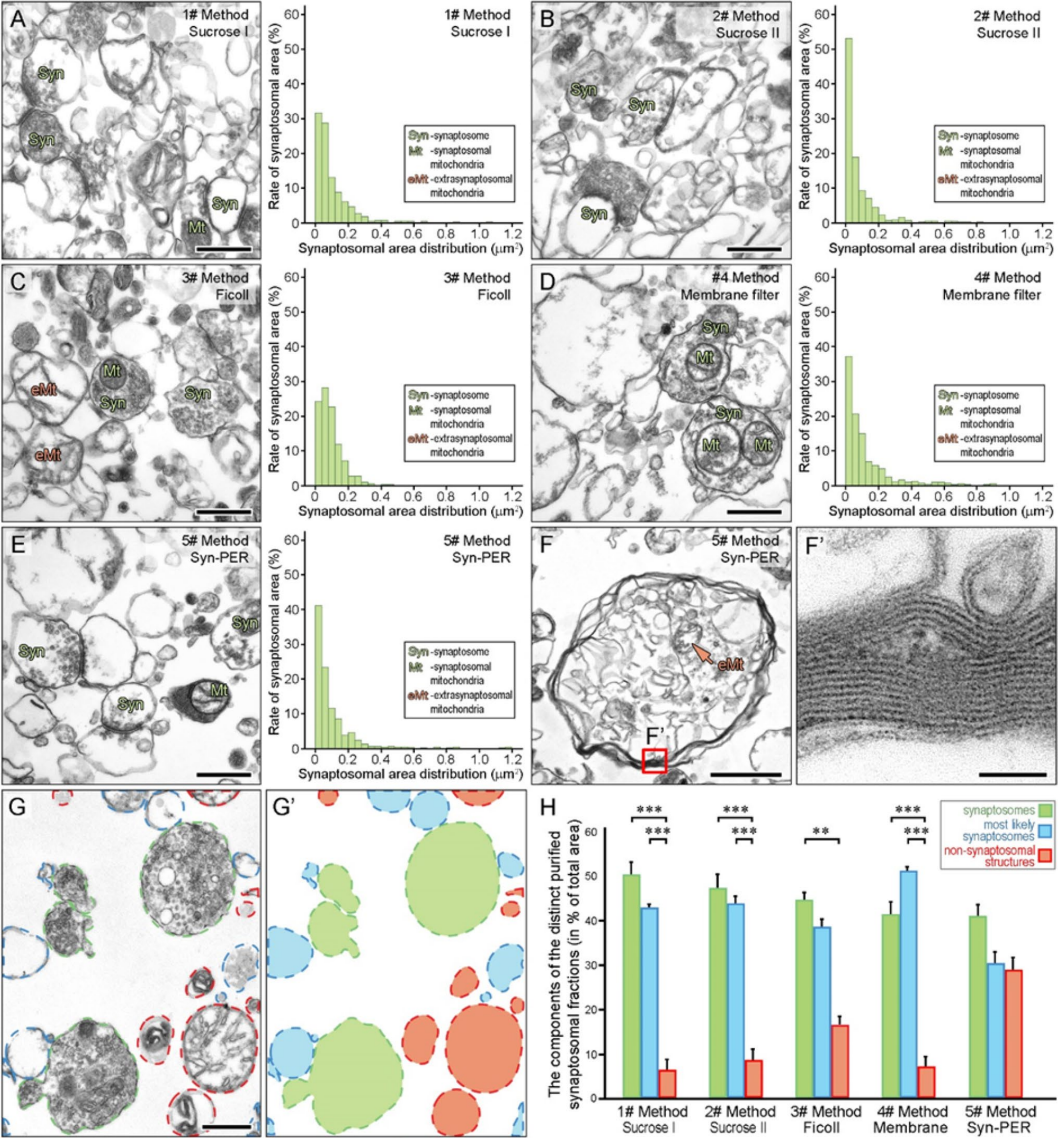
Validation and morphometric analysis of the purity of synaptosomal preparations with electron microscopy. **a**–**e**) Representative electron micrographs and the histograms of the synaptosomal size distribution are shown for all fractionation methods. **f** and **f**’ The left panel (**f**) shows an irregular-shaped multi-membranous structure in #5 Method preparation at low magnification and the right panel (**f**’) shows its magnified part. **g** and **g**’ Overview of the morphometric method. The left panel (**g**) shows the selection of the distinct components from an electron micrograph (green, certainly synaptosomes; blue, most likely synaptosomes; red, non-synaptosomal structures), while the one on the right-hand side (**g**’) presents colorized areas covered by the respective components. **h** Comparative analysis results on the areas of the subcellular components of the different synaptosomal preparations. **0.001 < *p* < 0.01; ***p < 0.001 (post hoc Mann–Whitney tests). Means ± SEM are shown for pooled data (12 images per sample) of each preparation. Scale bars: **a**–**e**, **g**: 0.5 µm, **f**: 1 µm, **f**’: 50 nm

Each sample contained intact synaptosomal structures in high abundance and three of them, the ones prepared with Method #1, #2, and #4 lacked nuclear or other intact cell organelles and debris (Fig. 1a–d), while the samples prepared with #3 Method and #5 Method showed a higher degree of contamination. It was apparent that the preparation procedure of #3 Method resulted in a sample that contains a high number of extrasynaptosomal mitochondria (Fig. 1c). The sample obtained following the #5 Method preparation showed irregular large-sized membrane or multi-membrane profiles containing unidentified cellular structures, exclusively characteristic of this fractionation method, and in some cases, extrasynaptosomal mitochondria (Fig. 1f, f’).

We quantified the purity of the preparations by using electron microscopic morphometry and compared them with each other by statistical analysis (Fig. 1g, g’). K–W test revealed a significant difference between the examined groups in their efficacy of isolation and degree of contamination. We compared the area distribution of the three subcellular groups obtained from each preparation with each other and analyzed the differences between the five preparation procedures as well (Fig. 1h). Post hoc M–W test showed that the area density values related to the certainly synaptosomal structures were statistically significantly higher compared to the non-synaptosomal structures in each preparation group (#1, #2, #4 Method: *p* < 0.001, #3 Method: 0.001 < *p* < 0.01) except the sample purified by #5 Method. Moreover, in the latter preparation, no significant differences were apparent between area densities of any of the components. This result can be accounted for by the observation that nearly 20% of the structures in this fraction are large-sized membranous non-synaptosomal components. Furthermore, we described statistically significantly higher area density values for most-likely synaptosomes in the preparations of #1, #2, and #4 Method (*p* < 0.001), but not in the cases of #3 and #5 Method, when compared to non-synaptosomal structures. Although the non-synaptosomal area density values are much higher in the samples processed according to #3 Method (16.60% ± 1.91%, means ± SEM) and #5 Method (28.78% ± 2.19%, means ± SEM), there were no statistically significant differences when we compared these samples to the other preparation groups (#1, #2, and #4 Method; 6.60% ± 0.77%, 8.80% ± 1.73%, and 7.34% ± 0.95%, respectively, means ± SEM).

In addition, we prepared histograms of the area size distribution of certainly synaptosomal structures, which showed similar characteristics in all preparations (Fig. 1a–e, right panels). The sample related to #2 Method contained the most small-sized synaptosomes (more than 50% of the synaptosomal area values were below 40 nm^2^) (Fig. 1b, right panel). Oppositely, #3 Method resulted in a preparation containing the less small-sized synaptosomes than in the other preparations (Fig. 1c, right panel). The sample prepared following #4 Method showed heterogeneity in the scale of larger size synaptosomes (Fig. 1d, right panel), which can be explained by the gentler centrifugation steps during the preparation procedure compared to the other methods.

### Western blot analysis of marker proteins in synaptosome samples

**Fig. 2:**
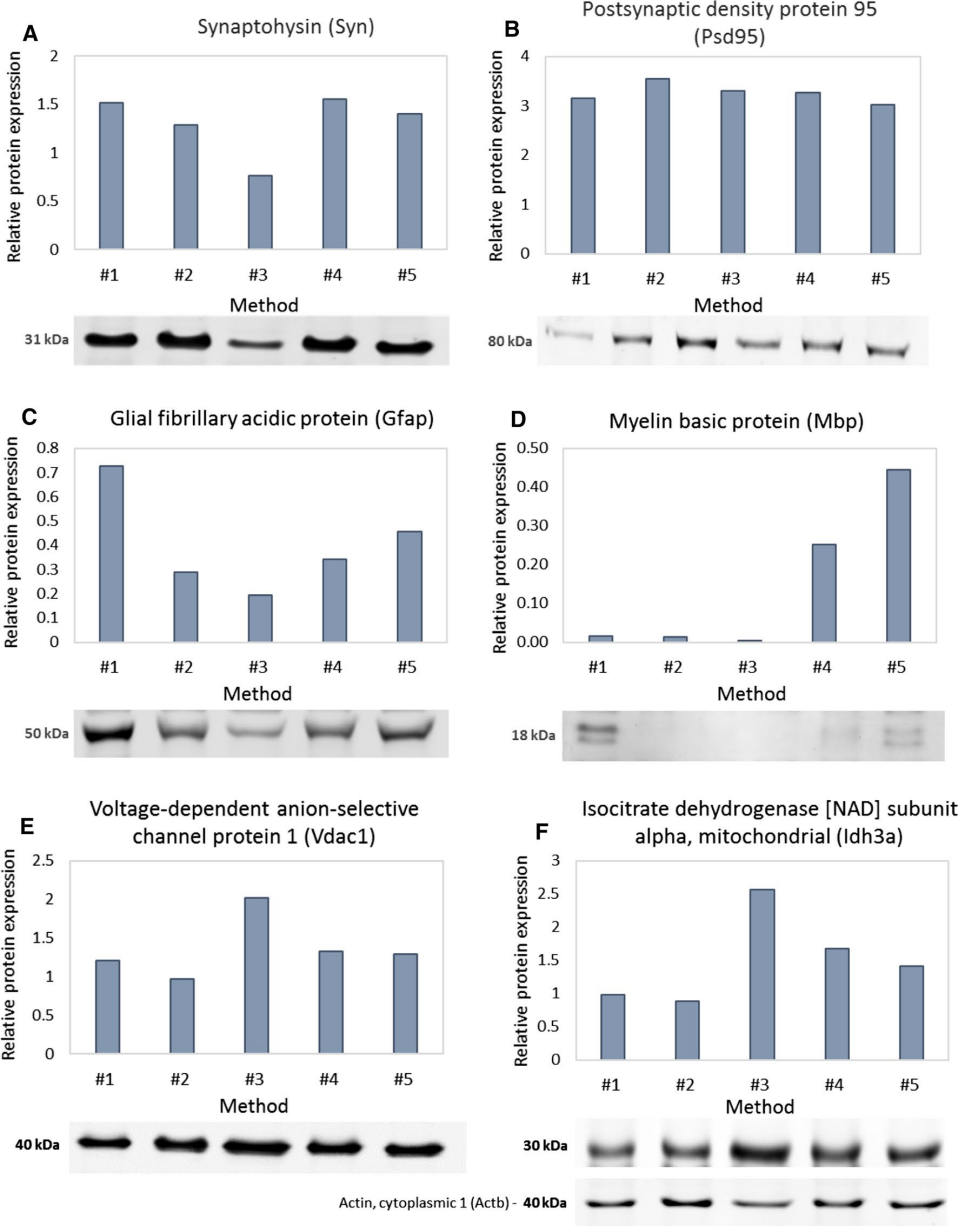
Western blot analysis of different synaptosomes preparations. Immunopositive bands and densitometric analysis are shown for synaptic- (**a**, **b**), glial- (**c**, **d**) and mitochondrial (**e**, **f**) marker proteins. Densitometric values in each sample are shown after normalization to the signal detected in the unfractionated cortical homogenate

One of the main goals of any subcellular fractionation approach in proteomics is to enrich the proteins in a given sample which would otherwise escape detection since their abundance in the whole tissue is lower than the detection limit characteristic for the applied analytical technique. A useful approach to monitor the enrichment of the fractionated organelle of interest is performing Western blot comparison with the whole tissue and examining enrichment in the level of well-accepted protein markers. In monitoring the efficacy of synaptosome isolation, pre- and postsynaptic marker proteins are of major importance as they directly reflect the presence of synaptic structures in the sample. In this study, we utilized the synaptic vesicle integral membrane glycoprotein synaptophysin as a presynaptic marker (Fig. 2a). Synaptic vesicles are trapped in the synaptosomes during the tissue homogenization process as the synaptic membranes reseal in the isoosmotic milieu and they can be observed as small circular objects with an almost identical diameter in electron microscopy images. As synaptic vesicles fuse with the synaptic membrane at the region of the active zone during the neurotransmitter exocytosis, the synaptophysin content of the sample has two sources. The protein can originate from the intact synaptic vesicles or from the synaptic plasma membrane-inserted pool. In our investigation, all samples showed an increased synaptophysin immunoreactivity (129.3–151.5%) in comparison with the whole tissue homogenate, except the samples prepared using a Ficoll-gradient according to #3 Method (76.3%). Postsynaptic density protein-95 (Psd-95), a major constituent of the postsynaptic density, responsible for clustering postsynaptic ion channels, receptors and signaling proteins were chosen as a postsynaptic marker protein. The postsynaptic density is tightly attached to the postsynaptic membrane and often remains bounded to the resealed presynaptic membrane and visible in electron microscopy images as an electron-dense thickening below the postsynaptic membrane. In accordance with our morphological observations, all samples showed a highly increased immunoreactivity for Psd-95 in the range of 301.7–354.9% (Fig. 2b). Therefore, although samples prepared via #3 Method showed no enrichment of the presynaptic marker, they were clearly enriched in the postsynaptic one. Thus, in this case, it is plausible that the synaptosome membranes were resealed properly during the tissue homogenization, but lost their integral content to some extent during the tissue disruption or the isolation process.

Another benefit of subcellular fractionation is the opportunity to deplete the sample from the unwanted contaminating proteins that reduce the complexity of the sample and enable the interpretation of the results of any proteomic experiment to be more straightforward. As most of the proteins present in a neuronal tissue are expressed by multiple cell types, it is important to ensure that the investigated protein pool in a proteomic experiment focusing on the synaptic proteome is neuron-derived. The heterogeneous population of glial cells comprises about half of the cells in the central nervous system in general (Bartheld et al. 2016). Astrocytes are assumed to be the most abundant glial cells in the cerebral cortex along with the oligodendrocytes, but unlike myelinating oligodendrocytes, they extend processes to the close proximity of synapses (Perea et al. 2009). Therefore, astrocytic structures (and functions) are hard to separate from the synapse, but synaptosome isolation gives a rational scope to physically remove them and deplete the astrocyte content of the tissue sample. We monitored the levels of the astrocyte-derived intermediate filament protein glial fibrillary acidic protein (Gfap) in the samples (Fig. 2c). All samples showed a lower immunoreactivity for the marker protein but the contamination levels were in a wide range (19.5–72.7%). Samples prepared with #3 Method showed the lowest, while those that fractionated with #1 Method showed the highest presence of astrocytic contamination. Oligodendrocyte contamination was followed in each sample by the analysis of the levels of myelin basic protein (Mbp) (Fig. 2d). Myelinating oligodendrocytes are located farther from synapses than astrocytes, creating the insulating myelin sheath along the axons and providing support for the neuron in terms of lactate, neurotrophic factors, and secreted exosomes. Oligodendrocytes ensheath dozens of adjacent axons in multiple layers, forming consecutive segments of the myelin sheath eventuating a decreased membrane capacitance and the saltatory propagation of action potentials. Mbp, being a major constituent of the myelin gives up to 30% of its protein content, has a prominent role in myelin compaction by binding opposing cytosolic surfaces of oligodendrocyte membranes (Boggs 2006). In our experiment, synaptosome isolation methods based on gradient centrifugation proved to be superior in the depletion of oligodendrocyte contamination. In the cases of samples obtained by #1, #2, and #3 Method, the detected Mbp immunoreactivity was 1.6%, 1.3%, and 0.5%, respectively, compared to the whole cortical tissue homogenate. Mbp immunoreactivity was extremely higher in samples isolated with #4 Method (25.2%) or #5 Method (44.6%). The high immunoreactivity of the samples obtained by using the commercially available kit is not surprising as around 20% of objects visible on electron micrographs were large-sized multi-membranous extrasynaptic debris clearly resembling the structure of the myelin sheath. The amount of mitochondria in a synaptosome sample can reflect the integrity of isolated nerve terminals and the efficacy of the isolation approach. Mitochondria are essential constituents of the functional nerve terminals and frequently visible on electron micrographs. However, mitochondria in the synaptosome preparation can originate from two sources. Intra-synaptosomal mitochondria are trapped in the synaptosome during the tissue homogenization and are inevitable components of the presynaptic transmission machinery. They provide energy supply to maintain the membrane potential, for information transfer, local protein synthesis, and have a peculiar proteome (Volgyi et al. 2015). On the other hand, extrasynaptic mitochondria can originate from the cell body of neurons or any glial cell types. The presence of extrasynaptosomal mitochondria renders it more difficult to interpret the proteomic results of any synapse-focused experiment; therefore it is a requirement to keep their proportion as low as possible. To measure the mitochondrial content of our synaptosome preparations, we investigated the level of mitochondrial matrix isocitrate dehydrogenase [NAD] subunit alpha (Idh3a) (Fig. 2e). Samples prepared with sucrose density-gradient centrifugation (#1 and #2 Method) showed a reduced immunoreactivity against Idh3a antibody, while the rest of the samples exhibited an elevated immunoreactivity. In agreement with our electron microscopy data, samples isolated with #3 Method had the highest mitochondrial content. Electron microscopy images of this fraction showed that a relatively high proportion of mitochondria are located outside the synaptosomes, and as their origin is uncertain, they should be considered as contamination. The higher immunoreactivity in samples prepared by #4 and #5 Method might arise from the fact that these samples contain relatively large synaptosomes and the isolation protocols lack centrifugal steps with high centrifugal forces producing elevated shear stress, thus, helping to maintain the native synaptosomal structures with inner synaptic mitochondria. Additionally, in the case of #5 Method, a higher presence of extrasynaptic mitochondria contamination, partially localized in the myelin sheath-originated membraneous debris, contributes to the elevated Idh3a immunoreactivity. Besides the Idh3a, we also evaluated the level of voltage-dependent anion-selective channel 1 (Vdac1), which has a dual localization: it forms an ion channel both in the outer mitochondrial membrane as well as the plasma membrane (Fig. 2f). Levels of Vdac1 showed a very similar pattern to the levels of Idh3a in all samples except for the one prepared with #1 Method. This latter sample showed a reduced level of Idh3a but an elevated level of Vdac1 compared to the whole tissue homogenate, which suggests its superior efficacy in synaptic membrane preservation and enrichment.

### Mass spectrometric analysis of the synaptic proteomes from the different preparations

The proteomes of the synaptosomes prepared with the five different isolation procedures were analyzed with mass spectrometry in a bottom-up approach. The protein content of the samples was enzymatically digested with trypsin, the generated peptides were separated with HPLC on a C18 stationary phase during a 132 min long gradient elution and the peptides were identified with a qToF mass spectrometer via electrospray ionization.

Several methodological opportunities exist to improve proteome coverage in proteomics studies. The number of identified peptides/proteins can be increased by using ultra-long elution times or performing sample prefractionation with multidimensional separation steps or by analyzing the sample in different *m/z* ranges during repeated MS measurements (Palma et al. 2012). In our study, we analyzed each sample in four consecutive runs. After the chromatographic separation, the peptides were analyzed in the 150–2200, 300–600, 650–850 and 850–2200 mass ranges, the MSMS^2^ spectra were merged and searched against the UniProt *Rattus norvegicus* dataset with MASCOT. The number of identified proteins in the samples were in the range of 1016–1196, among which 210–277 predicted to have transmembrane domains by the TMHMM software (Krogh et al. 2001) (#1 Method: 250/1028, #2 Method: 277/1196, #3 Method: 210/1016, #4 Method: 238/1128, #5 Method: 223/1103). A similar number of identified proteins indicates that our sample pretreatment and clean-up method is efficient for all isolation procedures and none of the samples contained contaminating detergents or impurities that interfere with the protein identification process.

**Fig. 3:**
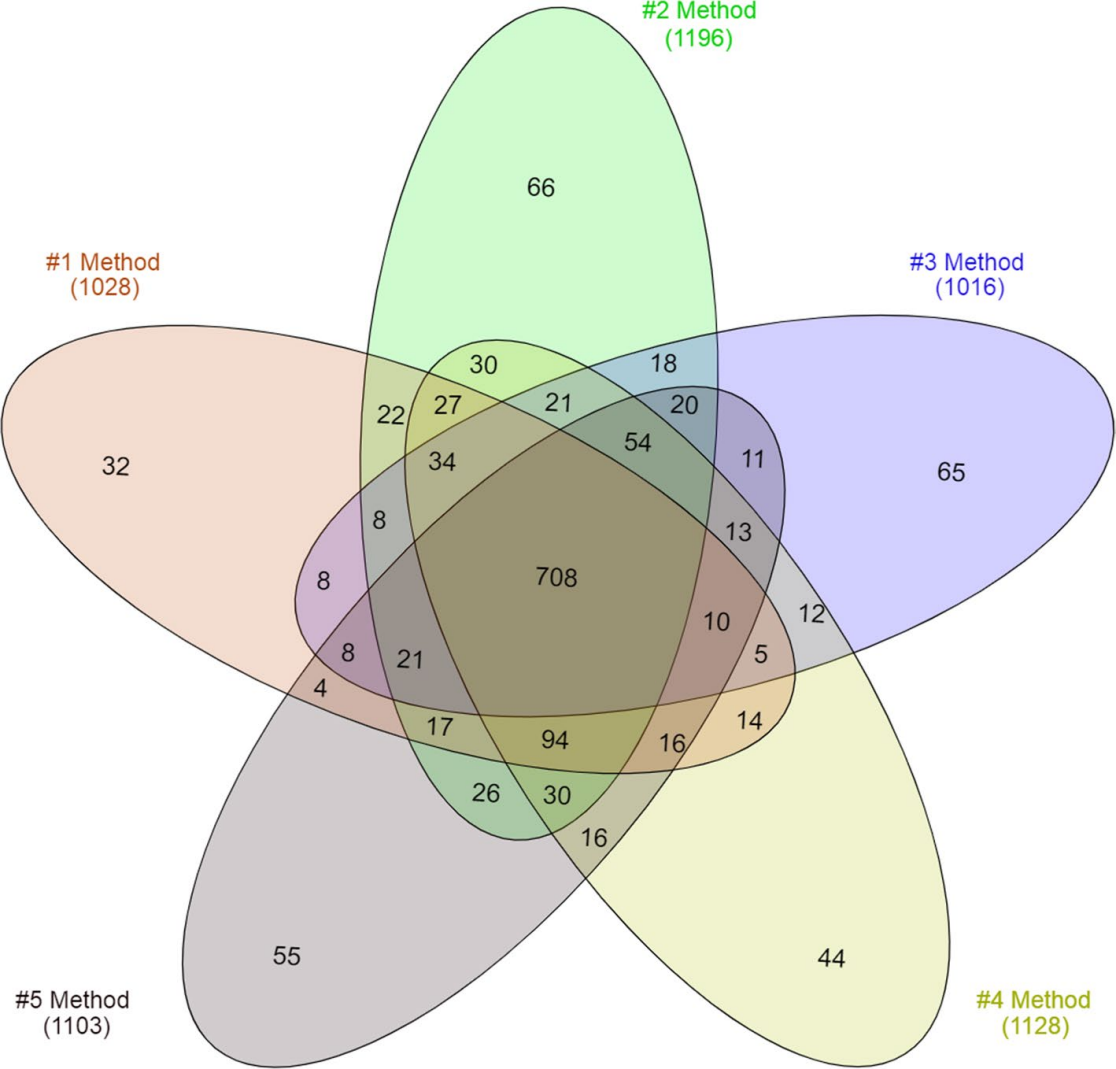
The numbers of common and unique proteins identified by LC–MS/MS in the synaptosome samples

The identified proteins having an overlapping presence in the samples and unique proteins characteristic only for a defined isolation method were analyzed with the InteractiVenn web-based tool (Fig. 3) (Heberle et al. 2015). Seven hundred and eight proteins were identified in all of the five samples (Supplementary Table 1), while 32–66 proteins were detectable in only one sample (#1 Method: 32, #2 Method: 66, #3 Method: 65, #4 Method: 44, #5 Method: 55) (Supplementary Table 2).

For the characterization of the proteomes from the five synaptosome samples, the open-source SynGO geneset analysis tool was used to describe the overrepresented synaptic terms comparing the identified proteins to the complete set of brain expressed proteins as a background (Koopmans et al. 2019). As synaptosomes being separated on the basis of their subcellular localization, the enrichment of terms in the „cellular compartment” class is notably informative (Supplementary Table 3). Among the most enriched terms „synapse”, „presynapse”, and „postsynapse” had the lowest p-values in all samples. In the point of all three terms, #2 Method outperformed the other methods having the highest gene counts, while #3 Method proved to contain the fewest synapse-specific proteins. The ratio of gene counts belonging to „presynapse” and „postsynapse” terms is highly similar in each sample. In the „biological process” class of ontology terms the „process in the synapse”, „process in the presynapse”, „synaptic vesicle cycle”, „synapse organization”, and „process in the postsynapse” were the ranking among the terms with the lowest p-values in the case of all samples (Supplementary Table 4). Analysis of the proteomes with open-source software GO::TermFinder revealed that the sample prepared by #3 Method has a relative high contamination of mitochondrial proteins (Boyle et al. 2004). Other cellular organelle-related terms apart from the neuron-specific ones in the top 20 were „mitochondrion” and „mitochondrial part” solely present in the case of sample isolated by #3 Method based on the presence of 298 and 219 related proteins in the described proteome, respectively (Supplementary Table 5). It is in agreement with our observation of the frequent presence of extrasynaptosomal mitochondria on the electron-microscopic images and with the high immunoreactivity of mitochondrial marker proteins.

Recently, SynaptomeDB has one of the largest collection of synaptic proteins, including 1886 genes and 4262 Gene Ontology terms, clustering them according to their sub-synaptic localization, as presynaptic, presynaptic active zone, synaptic vesicle, and postsynaptic proteins (Pirooznia et al. 2012). Comparison of the sub-synaptic localization of the proteins from the different synaptosome preparations using the SynaptomeDB classification system revealed a highly similar pattern in the classes of presynaptic, presynaptic active zone, and synaptic vesicle proteins. In the five synaptosome samples, 213–229 proteins proved to have presynaptic localisation, while 155–162 proteins were known as a constituent of the presynaptic active zone and 64–70 belonged to the group of synaptic vesicle proteins (Supplementary Table 6). A greater variance was observed in respect of postsynaptic components as samples contained 688–757 proteins of this class. The variance was contributed by the neuron-specific unique proteins identified in only one distinct sample, among which the vast majority belonged to the postsynaptic group (#1 Method: 15/32, #2 Method: 21/66, #3 Method: 15/65, #4 Method: 18/44, #5 Method: 15/55).

From the postsynaptic component of the synapse, synaptosomes contain nothing but a short segment of the postsynaptic membrane coupled to the postsynaptic density, therefore in this subcompartment membrane proteins are overrepresented. Accordingly, the relative occurrence of postsynaptic proteins in the samples coincided with the number of identified proteins having transmembrane domains. The sample prepared with #3 Method contained both membrane and postsynaptic proteins in the lowest number, while #2 Method provided both classes of proteins in the highest ratio in the sample.

## Conclusion

The neuroscience community utilizing synaptosomes as research objects go back on a long way and diverse in their purposes and methodological approaches. For the distinct scientific objectives and applied analytical techniques, different synaptosome isolation methods proved to be advantageous; not a single method exists, which is optimal for every research direction. In neuroproteomics experiments focusing on synapse biology, it is inevitable that the synaptosome sample has to contain enough protein material sufficient to downstream analysis, enriched in synaptic structures and proteins, and depleted from other cell types and contaminating subcellular organelles.

Electron microscopic images taken from the five samples clearly demonstrates that all of the isolation protocols we tested are useful to prepare and enrich synaptosomes from the nervous tissue. Synaptosomes dominate the structures in all-electron micrographs with characteristic synaptic features, such as the trapped synaptic vesicles and attached postsynaptic density. The enrichment of the synaptic structures was verified by the enhanced immunoreactivity for the synapse-specific marker proteins synaptophysin and Psd-95. Although, in our comparison, #3 Method had the tendency to lose its internal content leading to a reduced synaptophysin immunoreactivity, the enrichment of synaptosomes in the sample was unambiguous according to the morphometric findings. The proteome of the samples is largely overlapping: around two-third of the identified proteins are present in all of the samples. The majority of the detected proteins are known constituents of the synapse and a strikingly similar number of proteins belong to the group of the presynaptic, presynaptic active zone, and synaptic vesicle proteins. A greater variance was observed in the case of postsynaptic membrane proteins and proteins predicted to have a transmembrane domain. A vast majority of unique proteins present in only one of the samples are from the postsynaptic origin. The #3 Method underachieved in the enrichment of postsynaptic and transmembrane proteins, while #2 Method provided both groups of proteins in the highest ratio. Many explicit differences between the samples were observed in the case of contaminations. The sample prepared by #3 Method had the highest degree of contamination with extrasynaptic mitochondria, while the methods applying sucrose gradient centrifugation (#1 and #2 Method) were the most efficient in depletion of mitochondria located outside of the synaptosome. Despite the low efficacy in separating the synaptosomes from mitochondria, #3 Method outperformed the other methods in depletion of the sample from astrocytic contamination revealed by the low immunoreactivity for the astrocytic marker protein Gfap. An even more upfront contrast was apparent between the samples regarding oligodendrocyte contamination. While the level of the marker protein Mbp was even just above the detection limit in samples prepared by #1, #2, and #3 Method, the extent of myelin contamination in samples prepared by #4 and #5 Method was higher in orders of magnitude. Protein pools in all samples are highly enriched in synaptic proteins and presynaptic, synaptic vesicle, and postsynaptic proteins are represented in a relatively similar ratio hence we suggest that the sources and levels of contaminations should be considered as primary aspects considering the method of choice to isolate synaptosomes for proteomic experiments.

## Electronic supplementary material

Below is the link to the electronic supplementary material.

Supplementary file1 (DOCX 436 KB)

## Abbreviations

ACSF: Artificial cerebrospinal fluid
HEPES: 2-[4-(2-Hydroxyethyl)piperazin-1-yl]ethane sulfonic acid
CHAPS: 3-[(Cholamidopropyl)dimethylammonio]-1-propanesulfonate hydrate
DTT: 1,4-Dithiothreitol
SDS: Sodium dodecyl sulfate
PVDF: Polyvinylidene difluoride
TBS: Tris-buffered saline
MS: Mass spectrometry
LC–MS/MS: Liquid chromatography-tandem mass spectrometry
AcN: Acetonitrile
FA: Formic acid
TFA: Trifluoroacetic acid
CID: Collision-induced dissociation
PSD: Postsynaptic density
FACS: Fluorescence-activated cell sorter
SEM: Standard error of mean
