# Supplementary material for: Proteomic comparison of different synaptosome preparation procedures
Source: Amino Acids. 2020 Nov 19;52(11):1529–43. doi: 10.1007/s00726-020-02912-6 (PMC7695668; doi:10.1007/s00726-020-02912-6)
Supplement: Supplementary file 1 — Supplementary file1 (DOCX 436 KB) [file 726_2020_2912_MOESM1_ESM.docx]

Supplementary Table 1 List of the 708 common proteins identified with at least two unique peptides in all of the five synaptosome samples

| **Entry** | **Protein names** |
| --- | --- |
| P16086 | Spectrin alpha chain, non-erythrocytic 1 |
| P11442 | Clathrin heavy chain 1 |
| Q9QWN8 | Spectrin beta chain, non-erythrocytic 2 |
| P06687 | Sodium/potassium-transporting ATPase subunit alpha-3 |
| P06686 | Sodium/potassium-transporting ATPase subunit alpha-2 |
| P06685 | Sodium/potassium-transporting ATPase subunit alpha-1 |
| P38650 | Cytoplasmic dynein 1 heavy chain 1 |
| Q9QUL6 | Vesicle-fusing ATPase |
| P85108 | Tubulin beta-2A chain |
| Q3KRE8 | Tubulin beta-2B chain |
| P30427 | Plectin |
| P69897 | Tubulin beta-5 chain |
| P21575 | Dynamin-1 |
| Q6P9T8 | Tubulin beta-4B chain |
| Q4QRB4 | Tubulin beta-3 chain |
| P61765 | Syntaxin-binding protein 1 |
| P05708 | Hexokinase-1 |
| P10719 | ATP synthase subunit beta, mitochondrial |
| P15146 | Microtubule-associated protein 2 |
| Q63198 | Contactin-1 |
| P11505 | Plasma membrane calcium-transporting ATPase 1 |
| O88778 | Protein bassoon |
| P62944 | AP-2 complex subunit beta |
| P11506 | Plasma membrane calcium-transporting ATPase 2 |
| P63018 | Heat shock cognate 71 kDa protein |
| P11980 | Pyruvate kinase PKM |
| P34926 | Microtubule-associated protein 1A |
| Q9QUH6 | Ras/Rap GTPase-activating protein SynGAP |
| P68370 | Tubulin alpha-1A chain |
| P47942 | Dihydropyrimidinase-related protein 2 |
| Q9Z1P2 | Alpha-actinin-1 |
| P31596 | Excitatory amino acid transporter 2 |
| P63039 | 60 kDa heat shock protein, mitochondrial |
| Q6P9V9 | Tubulin alpha-1B chain |
| P18484 | AP-2 complex subunit alpha-2 |
| P15999 | ATP synthase subunit alpha, mitochondrial |
| Q9ER34 | Aconitate hydratase, mitochondrial |
| P13596 | Neural cell adhesion molecule 1 |
| Q5XIF6 | Tubulin alpha-4A chain |
| P15205 | Microtubule-associated protein 1B |
| Q9JLT0 | Myosin-10 |
| P11275 | Calcium/calmodulin-dependent protein kinase type II subunit alpha |
| P09951 | Synapsin-1 |
| P25286 | V-type proton ATPase 116 kDa subunit a isoform 1 |
| P05065 | Fructose-bisphosphate aldolase A |
| Q9QYF3 | Unconventional myosin-Va |
| P62815 | V-type proton ATPase subunit B, brain isoform |
| P59215 | Guanine nucleotide-binding protein G(o) subunit alpha |
| Q64542 | Plasma membrane calcium-transporting ATPase 4 |
| P21707 | Synaptotagmin-1 |
| Q63633 | Solute carrier family 12 member 5 |
| P11507 | Sarcoplasmic/endoplasmic reticulum calcium ATPase 2 |
| P00507 | Aspartate aminotransferase, mitochondrial |
| Q66HF1 | NADH-ubiquinone oxidoreductase 75 kDa subunit, mitochondrial |
| Q9JKS6 | Protein piccolo |
| Q05962 | ADP/ATP translocase 1 |
| P04797 | Glyceraldehyde-3-phosphate dehydrogenase |
| P31016 | Disks large homolog 4 |
| P50398 | Rab GDP dissociation inhibitor alpha |
| Q62950 | Dihydropyrimidinase-related protein 1 |
| P23565 | Alpha-internexin |
| P32551 | Cytochrome b-c1 complex subunit 2, mitochondrial |
| Q64568 | Plasma membrane calcium-transporting ATPase 3 |
| Q63537 | Synapsin-2 |
| P82995 | Heat shock protein HSP 90-alpha |
| Q9QXQ0 | Alpha-actinin-4 |
| P12839 | Neurofilament medium polypeptide |
| P34058 | Heat shock protein HSP 90-beta |
| P19527 | Neurofilament light polypeptide |
| Q05175 | Brain acid soluble protein 1 |
| P47819 | Glial fibrillary acidic protein |
| P13264 | Glutaminase kidney isoform, mitochondrial |
| Q09073 | ADP/ATP translocase 2 |
| O70511 | Ankyrin-3 |
| P04636 | Malate dehydrogenase, mitochondrial |
| P08413 | Calcium/calmodulin-dependent protein kinase type II subunit beta |
| P60881 | Synaptosomal-associated protein 25 |
| Q68FY0 | Cytochrome b-c1 complex subunit 1, mitochondrial |
| P53534 | Glycogen phosphorylase, brain form (Fragment) |
| Q920L2 | Succinate dehydrogenase [ubiquinone] flavoprotein subunit, mitochondrial |
| Q5XI78 | 2-oxoglutarate dehydrogenase, mitochondrial |
| P54290 | Voltage-dependent calcium channel subunit alpha-2/delta-1 |
| P07335 | Creatine kinase B-type |
| P07323 | Gamma-enolase |
| O35303 | Dynamin-1-like protein |
| P62260 | 14-3-3 protein epsilon |
| P42123 | L-lactate dehydrogenase B chain |
| Q9Z2L0 | Voltage-dependent anion-selective channel protein 1 |
| P04764 | Alpha-enolase |
| P54921 | Alpha-soluble NSF attachment protein |
| P63102 | 14-3-3 protein zeta/delta |
| Q9WU82 | Catenin beta-1 |
| P46462 | Transitional endoplasmic reticulum ATPase |
| P52303 | AP-1 complex subunit beta-1 |
| Q3KR86 | MICOS complex subunit Mic60 (Fragment) |
| P10824 | Guanine nucleotide-binding protein G(i) subunit alpha-1 |
| Q9WVC0 | Septin-7 |
| P97536 | Cullin-associated NEDD8-dissociated protein 1 |
| P63329 | Serine/threonine-protein phosphatase 2B catalytic subunit alpha isoform |
| Q63028 | Alpha-adducin |
| P97685 | Neurofascin |
| P85969 | Beta-soluble NSF attachment protein |
| Q64428 | Trifunctional enzyme subunit alpha, mitochondrial |
| Q9JI66 | Electrogenic sodium bicarbonate cotransporter 1 |
| P13233 | 2',3'-cyclic-nucleotide 3'-phosphodiesterase |
| Q62634 | Vesicular glutamate transporter 1 |
| Q5BK63 | NADH dehydrogenase [ubiquinone] 1 alpha subcomplex subunit 9, mitochondrial |
| P68255 | 14-3-3 protein theta |
| P11730 | Calcium/calmodulin-dependent protein kinase type II subunit gamma |
| P48721 | Stress-70 protein, mitochondrial |
| P04642 | L-lactate dehydrogenase A chain |
| P09117 | Fructose-bisphosphate aldolase C |
| Q9JJM9 | Septin-5 |
| Q62952 | Dihydropyrimidinase-related protein 3 |
| Q9ERH3 | WD repeat-containing protein 7 |
| P07340 | Sodium/potassium-transporting ATPase subunit beta-1 |
| P46101 | Dipeptidyl aminopeptidase-like protein 6 |
| D3ZHV2 | Microtubule-actin cross-linking factor 1 |
| P48500 | Triosephosphate isomerase |
| P32851 | Syntaxin-1A |
| P10687 | 1-phosphatidylinositol 4,5-bisphosphate phosphodiesterase beta-1 |
| P48037 | Annexin A6 |
| Q99NA5 | Isocitrate dehydrogenase [NAD] subunit alpha, mitochondrial |
| P19491 | Glutamate receptor 2 |
| Q794F9 | 4F2 cell-surface antigen heavy chain |
| P68511 | 14-3-3 protein eta |
| P97546 | Neuroplastin |
| Q63622 | Disks large homolog 2 |
| D3ZAA9 | MAGUK p55 subfamily member 2 |
| P06761 | Endoplasmic reticulum chaperone BiP |
| P16036 | Phosphate carrier protein, mitochondrial |
| Q08877 | Dynamin-3 |
| P84092 | AP-2 complex subunit mu |
| P35571 | Glycerol-3-phosphate dehydrogenase, mitochondrial |
| B3GNI6 | Septin-11 |
| P10860 | Glutamate dehydrogenase 1, mitochondrial |
| P54311 | Guanine nucleotide-binding protein G(I)/G(S)/G(T) subunit beta-1 |
| Q6P6V0 | Glucose-6-phosphate isomerase |
| P54313 | Guanine nucleotide-binding protein G(I)/G(S)/G(T) subunit beta-2 |
| P61265 | Syntaxin-1B |
| P35213 | 14-3-3 protein beta/alpha |
| Q02563 | Synaptic vesicle glycoprotein 2A |
| P47709 | Rabphilin-3A |
| P47858 | ATP-dependent 6-phosphofructokinase, muscle type |
| Q641Y2 | NADH dehydrogenase [ubiquinone] iron-sulfur protein 2, mitochondrial |
| Q9WTP0 | Band 4.1-like protein 1 |
| Q06647 | ATP synthase subunit O, mitochondrial |
| P68403 | Protein kinase C beta type |
| P61983 | 14-3-3 protein gamma |
| P02770 | Serum albumin |
| Q6P6R2 | Dihydrolipoyl dehydrogenase, mitochondrial |
| P16617 | Phosphoglycerate kinase 1 |
| Q9WTT6 | Guanine deaminase |
| P97846 | Contactin-associated protein 1 |
| Q80ZA5 | Sodium-driven chloride bicarbonate exchanger |
| Q63092 | CaM kinase-like vesicle-associated protein |
| O08838 | Amphiphysin |
| B0BNF1 | Septin-8 |
| Q63560 | Microtubule-associated protein 6 |
| P33124 | Long-chain-fatty-acid--CoA ligase 6 |
| Q07310 | Neurexin-3 |
| P0DP29 | Calmodulin-1 |
| P04897 | Guanine nucleotide-binding protein G(i) subunit alpha-2 |
| Q6PCU2 | V-type proton ATPase subunit E 1 |
| P81155 | Voltage-dependent anion-selective channel protein 2 |
| P47860 | ATP-dependent 6-phosphofructokinase, platelet type |
| P25809 | Creatine kinase U-type, mitochondrial |
| Q9Z0W5 | Protein kinase C and casein kinase substrate in neurons protein 1 |
| P19511 | ATP synthase F(0) complex subunit B1, mitochondrial |
| P05696 | Protein kinase C alpha type |
| P50399 | Rab GDP dissociation inhibitor beta |
| Q5XIN6 | Mitochondrial proton/calcium exchanger protein |
| B5DFN2 | S-adenosylhomocysteine hydrolase-like protein 1 |
| O35095 | Neurochondrin |
| P04775 | Sodium channel protein type 2 subunit alpha |
| Q2TA68 | Dynamin-like 120 kDa protein, mitochondrial |
| Q05695 | Neural cell adhesion molecule L1 |
| Q05764 | Beta-adducin |
| P20651 | Serine/threonine-protein phosphatase 2B catalytic subunit beta isoform |
| Q9QXY2 | SRC kinase signaling inhibitor 1 |
| Q63610 | Tropomyosin alpha-3 chain |
| P28480 | T-complex protein 1 subunit alpha |
| P12369 | cAMP-dependent protein kinase type II-beta regulatory subunit |
| P07936 | Neuromodulin |
| P08461 | Dihydrolipoyllysine-residue acetyltransferase component of pyruvate dehydrogenase complex, mitochondrial |
| P62632 | Elongation factor 1-alpha 2 |
| Q05140 | Clathrin coat assembly protein AP180 |
| P13221 | Aspartate aminotransferase, cytoplasmic |
| P19492 | Glutamate receptor 3 |
| P10111 | Peptidyl-prolyl cis-trans isomerase A |
| Q68FX0 | Isocitrate dehydrogenase [NAD] subunit beta, mitochondrial |
| Q66HA8 | Heat shock protein 105 kDa |
| P49432 | Pyruvate dehydrogenase E1 component subunit beta, mitochondrial |
| Q9JK11 | Reticulon-4 |
| P31399 | ATP synthase subunit d, mitochondrial |
| P61107 | Ras-related protein Rab-14 |
| P25113 | Phosphoglycerate mutase 1 |
| P29147 | D-beta-hydroxybutyrate dehydrogenase, mitochondrial |
| P48768 | Sodium/calcium exchanger 2 |
| P26284 | Pyruvate dehydrogenase E1 component subunit alpha, somatic form, mitochondrial |
| P02091 | Hemoglobin subunit beta-1 |
| Q64548 | Reticulon-1 |
| Q9Z1E1 | Flotillin-1 |
| P09606 | Glutamine synthetase |
| Q9Z214 | Homer protein homolog 1 |
| Q62910 | Synaptojanin-1 |
| P62138 | Serine/threonine-protein phosphatase PP1-alpha catalytic subunit |
| P11517 | Hemoglobin subunit beta-2 |
| Q4V7C7 | Actin-related protein 3 |
| P30835 | ATP-dependent 6-phosphofructokinase, liver type |
| Q6GMN2 | Brain-specific angiogenesis inhibitor 1-associated protein 2 |
| P97686 | Neuronal cell adhesion molecule |
| P18418 | Calreticulin |
| P12785 | Fatty acid synthase |
| P24942 | Excitatory amino acid transporter 1 |
| Q9JHY2 | Sideroflexin-3 |
| P31044 | Phosphatidylethanolamine-binding protein 1 |
| B2GV06 | Succinyl-CoA:3-ketoacid coenzyme A transferase 1, mitochondrial |
| Q561S0 | NADH dehydrogenase [ubiquinone] 1 alpha subcomplex subunit 10, mitochondrial |
| P63012 | Ras-related protein Rab-3A |
| Q63965 | Sideroflexin-1 |
| P62630 | Elongation factor 1-alpha 1 |
| P45592 | Cofilin-1 |
| P37805 | Transgelin-3 |
| Q5FVI6 | V-type proton ATPase subunit C 1 |
| Q62717 | Calcium-dependent secretion activator 1 |
| P50554 | 4-aminobutyrate aminotransferase, mitochondrial |
| Q5M7U6 | Actin-related protein 2 |
| Q6AXV4 | Sorting and assembly machinery component 50 homolog |
| P85970 | Actin-related protein 2/3 complex subunit 2 |
| Q9R1Z0 | Voltage-dependent anion-selective channel protein 3 ( |
| P67779 | Prohibitin |
| Q05683 | Glutamate decarboxylase 2 |
| P05508 | NADH-ubiquinone oxidoreductase chain 4 |
| P50137 | Transketolase |
| P61206 | ADP-ribosylation factor 3 |
| Q5XIH7 | Prohibitin-2 |
| O08839 | Myc box-dependent-interacting protein 1 |
| Q8VHF5 | Citrate synthase, mitochondrial |
| P62898 | Cytochrome c, somatic |
| P05712 | Ras-related protein Rab-2A |
| O35179 | Endophilin-A1 |
| Q9JHU0 | Dihydropyrimidinase-related protein 5 |
| P55161 | Nck-associated protein 1 |
| Q5PPJ9 | Endophilin-B2 |
| P62142 | Serine/threonine-protein phosphatase PP1-beta catalytic subunit |
| P05197 | Elongation factor 2 |
| Q6P502 | T-complex protein 1 subunit gamma |
| P70566 | Tropomodulin-2 |
| Q63564 | Synaptic vesicle glycoprotein 2B |
| Q00960 | Glutamate receptor ionotropic, NMDA 2B |
| Q6NYB7 | Ras-related protein Rab-1A |
| P29994 | Inositol 1,4,5-trisphosphate receptor type 1 |
| O35112 | CD166 antigen |
| P13638 | Sodium/potassium-transporting ATPase subunit beta-2 |
| Q5SGE0 | Leucine-rich PPR motif-containing protein, mitochondrial |
| O08662 | Phosphatidylinositol 4-kinase alpha |
| Q8R491 | EH domain-containing protein 3 |
| D4A6L0 | Probable G-protein coupled receptor 158 |
| Q6RJR6 | Reticulon-3 |
| Q5U300 | Ubiquitin-like modifier-activating enzyme 1 |
| Q75Q39 | Mitochondrial import receptor subunit TOM70 |
| P52873 | Pyruvate carboxylase, mitochondrial |
| P35565 | Calnexin |
| P35439 | Glutamate receptor ionotropic, NMDA 1 |
| Q66HD0 | Endoplasmin |
| Q64605 | Receptor-type tyrosine-protein phosphatase S |
| P15791 | Calcium/calmodulin-dependent protein kinase type II subunit delta |
| P01946 | Hemoglobin subunit alpha-1/2 |
| O88989 | Malate dehydrogenase, cytoplasmic |
| Q01062 | cGMP-dependent 3',5'-cyclic phosphodiesterase |
| P12368 | cAMP-dependent protein kinase type II-alpha regulatory subunit |
| P63319 | Protein kinase C gamma type |
| P35435 | ATP synthase subunit gamma, mitochondrial |
| Q62813 | Limbic system-associated membrane protein |
| O88600 | Heat shock 70 kDa protein 4 |
| Q5XIM9 | T-complex protein 1 subunit beta |
| P14668 | Annexin A5 |
| Q1WIM2 | Cell adhesion molecule 2 |
| P52481 | Adenylyl cyclase-associated protein 2 |
| Q8CFD0 | Sideroflexin-5 |
| P10536 | Ras-related protein Rab-1B |
| Q6AY30 | Saccharopine dehydrogenase-like oxidoreductase |
| Q99PD4 | Actin-related protein 2/3 complex subunit 1A |
| Q07266 | Drebrin |
| Q3T1K5 | F-actin-capping protein subunit alpha-2 |
| P19332 | Microtubule-associated protein tau |
| Q03555 | Gephyrin |
| P35704 | Peroxiredoxin-2 |
| P62762 | Visinin-like protein 1 |
| Q812E9 | Neuronal membrane glycoprotein M6-a |
| O08651 | D-3-phosphoglycerate dehydrogenase |
| P09527 | Ras-related protein Rab-7a |
| P97700 | Mitochondrial 2-oxoglutarate/malate carrier protein |
| O35244 | Peroxiredoxin-6 |
| P61589 | Transforming protein RhoA |
| Q8CFG5 | Voltage-dependent calcium channel subunit alpha-2/delta-3 |
| Q9Z327 | Synaptopodin |
| Q8K4Y5 | Leucine-rich glioma-inactivated protein 1 |
| P10960 | Prosaposin |
| P35281 | Ras-related protein Rab-10 |
| P28023 | Dynactin subunit 1 |
| Q9R063 | Peroxiredoxin-5, mitochondrial |
| P16638 | ATP-citrate synthase |
| P09812 | Glycogen phosphorylase, muscle form |
| P11598 | Protein disulfide-isomerase A3 |
| P70587 | Leucine-rich repeat-containing protein 7 |
| Q91Z79 | Liprin-alpha-3 |
| Q00981 | Ubiquitin carboxyl-terminal hydrolase isozyme L1 |
| P30009 | Myristoylated alanine-rich C-kinase substrate |
| O35353 | Guanine nucleotide-binding protein subunit beta-4 |
| P13086 | Succinate--CoA ligase [ADP/GDP-forming] subunit alpha, mitochondrial |
| P20788 | Cytochrome b-c1 complex subunit Rieske, mitochondrial |
| P39069 | Adenylate kinase isoenzyme 1 |
| P19627 | Guanine nucleotide-binding protein G(z) subunit alpha |
| P19490 | Glutamate receptor 1 |
| P11240 | Cytochrome c oxidase subunit 5A, mitochondrial |
| P00406 | Cytochrome c oxidase subunit 2 |
| Q4V8B0 | Oxidation resistance protein 1 |
| P19804 | Nucleoside diphosphate kinase B |
| Q62951 | Dihydropyrimidinase-related protein 4 |
| Q62718 | Neurotrimin |
| P37377 | Alpha-synuclein |
| Q8VBU2 | Protein NDRG2 |
| P21396 | Amine oxidase [flavin-containing] A |
| P62836 | Ras-related protein Rap-1A |
| O88917 | Adhesion G protein-coupled receptor L1 |
| Q9Z2S9 | Flotillin-2 |
| P62747 | Rho-related GTP-binding protein RhoB |
| P82471 | Guanine nucleotide-binding protein G(q) subunit alpha |
| P85834 | Elongation factor Tu, mitochondrial |
| P10888 | Cytochrome c oxidase subunit 4 isoform 1, mitochondrial |
| Q62768 | Protein unc-13 homolog A |
| Q6Q629 | Inactive dipeptidyl peptidase 10 |
| Q5BKC9 | Ephexin-1 |
| P97610 | Synaptotagmin-12 |
| P31422 | Metabotropic glutamate receptor 3 |
| Q6AXT5 | Ras-related protein Rab-21 |
| P52296 | Importin subunit beta-1 |
| Q66HA6 | ADP-ribosylation factor-like protein 8B |
| Q568Z9 | Phytanoyl-CoA hydroxylase-interacting protein |
| P97924 | Kalirin |
| P49803 | Regulator of G-protein signaling 7 |
| Q62936 | Disks large homolog 3 |
| P0DJJ3 | SH3-containing GRB2-like protein 3-interacting protein 1 |
| P47875 | Cysteine and glycine-rich protein 1 |
| P04692 | Tropomyosin alpha-1 chain |
| Q4FZY0 | EF-hand domain-containing protein D2 |
| P0C6S7 | Ankyrin repeat and sterile alpha motif domain-containing protein 1B |
| Q9JLU4 | SH3 and multiple ankyrin repeat domains protein 3 |
| P01830 | Thy-1 membrane glycoprotein |
| P85515 | Alpha-centractin |
| P97710 | Tyrosine-protein phosphatase non-receptor type substrate 1 |
| P14408 | Fumarate hydratase, mitochondrial |
| Q63754 | Beta-synuclein |
| P62824 | Ras-related protein Rab-3C |
| Q5U316 | Ras-related protein Rab-35 |
| Q6PST4 | Atlastin-1 |
| Q5RKI0 | WD repeat-containing protein 1 |
| Q08163 | Adenylyl cyclase-associated protein 1 |
| Q5RJQ4 | NAD-dependent protein deacetylase sirtuin-2 |
| P17764 | Acetyl-CoA acetyltransferase, mitochondrial |
| P0C1X8 | AP2-associated protein kinase 1 |
| P85845 | Fascin |
| P31977 | Ezrin |
| O55012 | Phosphatidylinositol-binding clathrin assembly protein |
| Q1WIM3 | Cell adhesion molecule 3 |
| Q9QX69 | Glutathione S-transferase LANCL1 |
| Q05982 | Nucleoside diphosphate kinase A |
| P63331 | Serine/threonine-protein phosphatase 2A catalytic subunit alpha isoform |
| B2RYW9 | Fumarylacetoacetate hydrolase domain-containing protein 2 |
| P60203 | Myelin proteolipid protein |
| Q6AYH5 | Dynactin subunit 2 |
| Q99ML5 | Prenylcysteine oxidase |
| Q9JID2 | Guanine nucleotide-binding protein subunit alpha-11 |
| Q6P7R8 | Very-long-chain 3-oxoacyl-CoA reductase |
| P04905 | Glutathione S-transferase Mu 1 |
| Q5XIF3 | NADH dehydrogenase [ubiquinone] iron-sulfur protein 4, mitochondrial |
| P24587 | A-kinase anchor protein 5 |
| Q5BJU7 | Wiskott-Aldrich syndrome protein family member 1 |
| P08009 | Glutathione S-transferase Yb-3 |
| Q9WVB1 | Ras-related protein Rab-6A |
| Q62847 | Gamma-adducin |
| Q63372 | Neurexin-1 |
| Q62915 | Peripheral plasma membrane protein CASK |
| Q9WU34 | Neuronal-specific septin-3 |
| F1M0Z1 | Triple functional domain protein |
| P16446 | Phosphatidylinositol transfer protein alpha isoform |
| P35280 | Ras-related protein Rab-8A |
| Q924N5 | Long-chain-fatty-acid--CoA ligase ACSBG1 |
| P08082 | Clathrin light chain B |
| O08873 | MAP kinase-activating death domain protein |
| P41565 | Isocitrate dehydrogenase [NAD] subunit gamma 1, mitochondrial |
| Q5XI32 | F-actin-capping protein subunit beta |
| P02688 | Myelin basic protein |
| O35796 | Complement component 1 Q subcomponent-binding protein, mitochondrial |
| Q62658 | Peptidyl-prolyl cis-trans isomerase FKBP1A |
| P62882 | Guanine nucleotide-binding protein subunit beta-5 |
| P61227 | Ras-related protein Rap-2b |
| Q920Q0 | Paralemmin-1 |
| Q9WV48 | SH3 and multiple ankyrin repeat domains protein 1 |
| Q62696 | Disks large homolog 1 |
| P63079 | Gamma-aminobutyric acid receptor subunit beta-3 |
| P19234 | NADH dehydrogenase [ubiquinone] flavoprotein 2, mitochondrial |
| Q01205 | Dihydrolipoyllysine-residue succinyltransferase component of 2-oxoglutarate dehydrogenase complex, mitochondrial |
| Q8R431 | Monoglyceride lipase |
| Q5XI73 | Rho GDP-dissociation inhibitor 1 |
| P62963 | Profilin-1 |
| Q91ZN1 | Coronin-1A |
| Q6P0K8 | Junction plakoglobin |
| Q91Y81 | Septin-2 |
| Q8CFN2 | Cell division control protein 42 homolog |
| Q9QYU4 | Ketimine reductase mu-crystallin |
| Q80W89 | NADH dehydrogenase [ubiquinone] 1 alpha subcomplex subunit 11 |
| O35964 | Endophilin-A2 |
| P07895 | Superoxide dismutase [Mn], mitochondrial |
| O35331 | Pyridoxal kinase |
| O35814 | Stress-induced-phosphoprotein 1 |
| Q6RUV5 | Ras-related C3 botulinum toxin substrate 1 |
| P50408 | V-type proton ATPase subunit F |
| P63086 | Mitogen-activated protein kinase 1 |
| Q63362 | NADH dehydrogenase [ubiquinone] 1 alpha subcomplex subunit 5 |
| Q62889 | Neuroligin-3 |
| P32736 | Opioid-binding protein/cell adhesion molecule |
| Q5XHZ0 | Heat shock protein 75 kDa, mitochondrial |
| Q60587 | Trifunctional enzyme subunit beta, mitochondrial |
| O35509 | Ras-related protein Rab-11B |
| Q63083 | Nucleobindin-1 |
| Q924S5 | Lon protease homolog, mitochondrial |
| P14669 | Annexin A3 |
| Q9Z0J8 | Neuronal growth regulator 1 |
| P19643 | Amine oxidase [flavin-containing] B |
| Q641Z6 | EH domain-containing protein 1 |
| B2GV54 | Neutral cholesterol ester hydrolase 1 |
| P04785 | Protein disulfide-isomerase |
| P11884 | Aldehyde dehydrogenase, mitochondrial |
| Q9WU70 | Syntaxin-binding protein 5 |
| P08081 | Clathrin light chain A |
| O88767 | Protein/nucleic acid deglycase DJ-1 |
| P29419 | ATP synthase subunit e, mitochondrial |
| P04631 | Protein S100-B |
| P63045 | Vesicle-associated membrane protein 2 |
| Q3MIE4 | Synaptic vesicle membrane protein VAT-1 homolog |
| Q7TPB1 | T-complex protein 1 subunit delta |
| P07171 | Calbindin |
| P24368 | Peptidyl-prolyl cis-trans isomerase B |
| P68182 | cAMP-dependent protein kinase catalytic subunit beta |
| P10818 | Cytochrome c oxidase subunit 6A1, mitochondrial |
| P14604 | Enoyl-CoA hydratase, mitochondrial |
| P23978 | Sodium- and chloride-dependent GABA transporter 1 |
| P26772 | 10 kDa heat shock protein, mitochondrial |
| P84083 | ADP-ribosylation factor 5 |
| O70351 | 3-hydroxyacyl-CoA dehydrogenase type-2 |
| P62483 | Voltage-gated potassium channel subunit beta-2 |
| B0BND0 | Ectonucleotide pyrophosphatase/phosphodiesterase family member 6 |
| P08050 | Gap junction alpha-1 protein |
| O35274 | Neurabin-2 |
| P63081 | V-type proton ATPase 16 kDa proteolipid subunit |
| P31421 | Metabotropic glutamate receptor 2 |
| M0RC99 | Ras-related protein Rab-5A |
| P38652 | Phosphoglucomutase-1 |
| Q64560 | Tripeptidyl-peptidase 2 |
| P97612 | Fatty-acid amide hydrolase 1 |
| P03889 | NADH-ubiquinone oxidoreductase chain 1 |
| O35987 | NSFL1 cofactor p47 |
| Q62848 | ADP-ribosylation factor GTPase-activating protein 1 |
| D4A1R8 | Copine-1 |
| Q63617 | Hypoxia up-regulated protein 1 |
| Q63081 | Protein disulfide-isomerase A6 |
| Q5FVJ0 | Protein RUFY3 |
| P60905 | DnaJ homolog subfamily C member 5 |
| P18508 | Gamma-aminobutyric acid receptor subunit gamma-2 |
| P53678 | AP-3 complex subunit mu-2 |
| Q07647 | Solute carrier family 2, facilitated glucose transporter member 3 |
| Q63377 | Sodium/potassium-transporting ATPase subunit beta-3 |
| P31662 | Sodium-dependent neutral amino acid transporter SLC6A17 |
| P21913 | Succinate dehydrogenase [ubiquinone] iron-sulfur subunit, mitochondrial |
| P62744 | AP-2 complex subunit sigma |
| O35116 | Catenin delta-2 (Fragment) |
| Q4KM73 | UMP-CMP kinase |
| Q75Q40 | Mitochondrial import receptor subunit TOM40 homolog |
| O08875 | Serine/threonine-protein kinase DCLK1 |
| Q9Z272 | ARF GTPase-activating protein GIT1 |
| O88871 | Gamma-aminobutyric acid type B receptor subunit 2 |
| P00787 | Cathepsin B |
| Q7TQ16 | Cytochrome b-c1 complex subunit 8 |
| P35465 | Serine/threonine-protein kinase PAK 1 |
| O35458 | Vesicular inhibitory amino acid transporter |
| Q6P7Q4 | Lactoylglutathione lyase |
| P13803 | Electron transfer flavoprotein subunit alpha, mitochondrial |
| F1LP90 | Misshapen-like kinase 1 |
| P56558 | UDP-N-acetylglucosamine--peptide N-acetylglucosaminyltransferase 110 kDa subunit |
| Q5EB77 | Ras-related protein Rab-18 |
| P31647 | Sodium- and chloride-dependent GABA transporter 3 |
| O88588 | Phosphofurin acidic cluster sorting protein 1 |
| Q9EPC6 | Profilin-2 |
| Q5HZV9 | Protein phosphatase 1 regulatory subunit 7 |
| Q4V8I7 | Volume-regulated anion channel subunit LRRC8A |
| P63004 | Platelet-activating factor acetylhydrolase IB subunit alpha |
| P56571 | ES1 protein homolog, mitochondrial |
| P0C5X8 | Protein tweety homolog 1 |
| Q4QQT4 | Serine/threonine-protein phosphatase 2A 65 kDa regulatory subunit A beta isoform |
| Q62747 | Synaptotagmin-7 |
| Q4FZT0 | Stomatin-like protein 2, mitochondrial |
| Q5FVI4 | Cell cycle exit and neuronal differentiation protein 1 |
| P63041 | Complexin-1 |
| Q68FS2 | COP9 signalosome complex subunit 4 |
| P07825 | Synaptophysin |
| P54287 | Voltage-dependent L-type calcium channel subunit beta-3 |
| Q62812 | Myosin-9 |
| P26453 | Basigin |
| Q9Z2F5 | C-terminal-binding protein 1 |
| Q01986 | Dual specificity mitogen-activated protein kinase kinase 1 |
| B0K020 | CDGSH iron-sulfur domain-containing protein 1 |
| Q9Z1Y3 | Cadherin-2 |
| Q64559 | Cytosolic acyl coenzyme A thioester hydrolase |
| Q63716 | Peroxiredoxin-1 |
| P40112 | Proteasome subunit beta type-3 |
| P97849 | Long-chain fatty acid transport protein 1 |
| Q78P75 | Dynein light chain 2, cytoplasmic |
| P84087 | Complexin-2 |
| Q9QZR6 | Septin-9 |
| O55043 | Rho guanine nucleotide exchange factor 7 |
| P62959 | Histidine triad nucleotide-binding protein 1 |
| P56574 | Isocitrate dehydrogenase [NADP], mitochondrial |
| Q4V8B7 | Inactive hydroxysteroid dehydrogenase-like protein 1 |
| Q4V7D2 | Protein rogdi homolog |
| P51650 | Succinate-semialdehyde dehydrogenase, mitochondrial |
| Q9WVK7 | Hydroxyacyl-coenzyme A dehydrogenase, mitochondrial |
| Q9JHL4 | Drebrin-like protein |
| Q62888 | Neuroligin-2 |
| O35764 | Neuronal pentraxin receptor |
| A1L1I3 | Numb-like protein |
| Q9QYJ6 | cAMP and cAMP-inhibited cGMP 3',5'-cyclic phosphodiesterase 10A |
| Q6QIX3 | Zinc transporter 3 |
| B2GUZ5 | F-actin-capping protein subunit alpha-1 |
| Q765A7 | GPI inositol-deacylase |
| Q5U318 | Astrocytic phosphoprotein PEA-15 |
| Q99MZ8 | LIM and SH3 domain protein 1 |
| Q80Z30 | Protein phosphatase 1E ( |
| P85973 | Purine nucleoside phosphorylase |
| Q01728 | Sodium/calcium exchanger 1 |
| Q04970 | GTPase Nras |
| B2RYG6 | Ubiquitin thioesterase OTUB1 |
| P11951 | Cytochrome c oxidase subunit 6C-2 |
| P13852 | Major prion protein |
| Q9Z0U4 | Gamma-aminobutyric acid type B receptor subunit 1 |
| O35867 | Neurabin-1 |
| Q6AY84 | Secernin-1 |
| Q6DGG0 | Peptidyl-prolyl cis-trans isomerase D |
| P11232 | Thioredoxin |
| Q6UPE1 | Electron transfer flavoprotein-ubiquinone oxidoreductase, mitochondrial |
| Q5BJS7 | Copine-9 |
| Q64119 | Myosin light polypeptide 6 |
| P80254 | D-dopachrome decarboxylase |
| P29266 | 3-hydroxyisobutyrate dehydrogenase, mitochondrial |
| Q63569 | 26S proteasome regulatory subunit 6A |
| P11348 | Dihydropteridine reductase |
| Q62609 | Noelin |
| Q9ES21 | Phosphatidylinositide phosphatase SAC1 |
| P47863 | Aquaporin-4 |
| Q04400 | Adenylate cyclase type 5 |
| P12075 | Cytochrome c oxidase subunit 5B, mitochondrial |
| P62703 | 40S ribosomal protein S4, X isoform |
| Q5EB81 | NADH-cytochrome b5 reductase 1 |
| Q7M0E3 | Destrin |
| P10760 | Adenosylhomocysteinase |
| P17105 | Inositol-trisphosphate 3-kinase A |
| G3V7P1 | Syntaxin-12 |
| Q9Z270 | Vesicle-associated membrane protein-associated protein A |
| Q9EPH2 | MARCKS-related protein |
| Q6PDU7 | ATP synthase subunit g, mitochondrial |
| P97852 | Peroxisomal multifunctional enzyme type 2 |
| Q9JJW3 | ATP synthase membrane subunit DAPIT, mitochondrial |
| P07632 | Superoxide dismutase [Cu-Zn] |
| P27791 | cAMP-dependent protein kinase catalytic subunit alpha |
| Q9JM53 | Apoptosis-inducing factor 1, mitochondrial |
| Q5PQN0 | Neurocalcin-delta |
| P04904 | Glutathione S-transferase alpha-3 |
| P20171 | GTPase HRas |
| P22062 | Protein-L-isoaspartate(D-aspartate) O-methyltransferase |
| O08700 | Vacuolar protein sorting-associated protein 45 |
| P11661 | NADH-ubiquinone oxidoreductase chain 5 |
| P54690 | Branched-chain-amino-acid aminotransferase, cytosolic |
| Q66HL2 | Src substrate cortactin |
| P36876 | Serine/threonine-protein phosphatase 2A 55 kDa regulatory subunit B alpha isoform |
| Q5M7A7 | CB1 cannabinoid receptor-interacting protein 1 |
| P27139 | Carbonic anhydrase 2 |
| P57113 | Maleylacetoacetate isomerase |
| Q8VD52 | Pyridoxal phosphate phosphatase |
| P51146 | Ras-related protein Rab-4B |
| P04906 | Glutathione S-transferase P |
| P35171 | Cytochrome c oxidase subunit 7A2, mitochondrial |
| F1LQX4 | Rho GTPase-activating protein 44 |
| P47728 | Calretinin |
| P38718 | Mitochondrial pyruvate carrier 2 |
| Q792I0 | Protein lin-7 homolog C |
| P05371 | Clusterin |
| Q80WF4 | Transmembrane protein 132A |
| D3ZAF6 | ATP synthase subunit f, mitochondrial |
| Q704E8 | ATP-binding cassette sub-family B member 7, mitochondrial |
| P63322 | Ras-related protein Ral-A |
| P25235 | Dolichyl-diphosphooligosaccharide--protein glycosyltransferase subunit 2 |
| P52555 | Endoplasmic reticulum resident protein 29 |
| B5DF41 | Syntaphilin |
| Q6PEC4 | S-phase kinase-associated protein 1 |
| Q6Q7Y5 | Guanine nucleotide-binding protein subunit alpha-13 |
| Q5RKI1 | Eukaryotic initiation factor 4A-II |
| P21571 | ATP synthase-coupling factor 6, mitochondrial |
| Q9Z1B2 | Glutathione S-transferase Mu 5 |
| Q8K1M8 | BMP/retinoic acid-inducible neural-specific protein 2 |
| Q66H15 | Regulator of microtubule dynamics protein 3 |
| P69682 | Adaptin ear-binding coat-associated protein 1 |
| P35284 | Ras-related protein Rab-12 |
| Q9JJK1 | Neuronal membrane glycoprotein M6-b |
| G3V7W1 | Programmed cell death protein 6 |
| P70483 | Striatin |
| Q6AXX6 | Peroxiredoxin-like 2A |
| Q9Z0V6 | Thioredoxin-dependent peroxide reductase, mitochondrial |
| P05426 | 60S ribosomal protein L7 |
| Q566R0 | Acyl-coenzyme A thioesterase THEM4 |
| P12346 | Serotransferrin |
| B1WBW4 | Armadillo repeat-containing protein 10 |
| P11030 | Acyl-CoA-binding protein |
| P84817 | Mitochondrial fission 1 protein |
| P62083 | 40S ribosomal protein S7 |
| Q5XIE6 | 3-hydroxyisobutyryl-CoA hydrolase, mitochondrial |
| D3ZQG6 | Tripartite motif-containing protein 2 |
| P51635 | Aldo-keto reductase family 1 member A1 |
| O35094 | Mitochondrial import inner membrane translocase subunit TIM44 |
| P84076 | Neuron-specific calcium-binding protein hippocalcin |
| Q9EPH8 | Polyadenylate-binding protein 1 |
| P18421 | Proteasome subunit beta type-1 |
| Q5BK32 | FAS-associated factor 2 |
| Q3KRE0 | ATPase family AAA domain-containing protein 3 |
| Q7TT47 | Paraplegin |
| Q920J4 | Thioredoxin-like protein 1 |
| Q5XIT1 | Microtubule-associated protein RP/EB family member 3 |
| P63142 | Potassium voltage-gated channel subfamily A member 2 |
| O70277 | Tripartite motif-containing protein 3 |
| Q641Y0 | Dolichyl-diphosphooligosaccharide--protein glycosyltransferase 48 kDa subunit |
| P27605 | Hypoxanthine-guanine phosphoribosyltransferase |
| P62909 | 40S ribosomal protein S3 |
| Q8R500 | Mitofusin-2 |
| Q99JD4 | CLIP-associating protein 2 |
| Q07009 | Calpain-2 catalytic subunit |
| Q505J6 | Mitochondrial glutamate carrier 2 |
| P17220 | Proteasome subunit alpha type-2 |
| Q5I0D5 | Phospholysine phosphohistidine inorganic pyrophosphate phosphatase |
| P62994 | Growth factor receptor-bound protein 2 |
| Q5QD51 | A-kinase anchor protein 12 |
| Q52KK3 | Solute carrier family 25 member 51 |
| P02401 | 60S acidic ribosomal protein P2 |
| P30904 | Macrophage migration inhibitory factor |
| A2RUW1 | Toll-interacting protein |
| O35567 | Bifunctional purine biosynthesis protein PURH |
| P12001 | 60S ribosomal protein L18 |
| P18666 | Myosin regulatory light chain 12B |
| Q99MZ4 | Glutathione hydrolase 7 |
| Q9Z339 | Glutathione S-transferase omega-1 |
| A0JPJ7 | Obg-like ATPase 1 |
| D4A7N1 | MICOS complex subunit Mic25 |
| P20070 | NADH-cytochrome b5 reductase 3 |
| Q66HF8 | Aldehyde dehydrogenase X, mitochondrial |
| P19945 | 60S acidic ribosomal protein P0 |
| P36972 | Adenine phosphoribosyltransferase |
| B0BNM1 | NAD(P)H-hydrate epimerase |
| P21533 | 60S ribosomal protein L6 |
| Q64057 | Alpha-aminoadipic semialdehyde dehydrogenase |
| P32089 | Tricarboxylate transport protein, mitochondrial |
| O08557 | N(G),N(G)-dimethylarginine dimethylaminohydrolase 1 |
| Q5RKI8 | ATP-binding cassette sub-family B member 8, mitochondrial |
| B2RZ78 | Vacuolar protein sorting-associated protein 29 |
| Q6AY55 | Dephospho-CoA kinase domain-containing protein |
| Q9EQX9 | Ubiquitin-conjugating enzyme E2 N |
| O54975 | Xaa-Pro aminopeptidase 1 |
| Q5U2Z3 | Nucleosome assembly protein 1-like 4 |
| Q62876 | Synaptogyrin-1 |
| O88339 | Epsin-1 |
| P27274 | CD59 glycoprotein |
| Q5RJR8 | Leucine-rich repeat-containing protein 59 |
| P07943 | Aldo-keto reductase family 1 member B1 |
| Q8VI04 | Isoaspartyl peptidase/L-asparaginase |
| P62168 | Neuronal calcium sensor 1 |
| P50878 | 60S ribosomal protein L4 |
| Q4G017 | Nischarin |
| Q9ERQ6 | Chondroitin sulfate proteoglycan 5 |
| Q5XIT9 | Methylcrotonoyl-CoA carboxylase beta chain, mitochondrial |
| P07153 | Dolichyl-diphosphooligosaccharide--protein glycosyltransferase subunit 1 |
| P18420 | Proteasome subunit alpha type-1 |
| Q75Q41 | Mitochondrial import receptor subunit TOM22 homolog |
| O35264 | Platelet-activating factor acetylhydrolase IB subunit beta |
| Q8K3P6 | Calcium-binding mitochondrial carrier protein SCaMC-2 |
| P30839 | Aldehyde dehydrogenase family 3 member A2 |
| P15087 | Carboxypeptidase E |
| Q64640 | Adenosine kinase |
| P63245 | Receptor of activated protein C kinase 1 |
| Q64536 | [Pyruvate dehydrogenase (acetyl-transferring)] kinase isozyme 2, mitochondrial |
| Q2LC84 | Protein numb homolog |
| P62250 | 40S ribosomal protein S16 |
| Q68FR9 | Elongation factor 1-delta |
| Q6P6Q9 | Calcium uptake protein 1, mitochondrial |
| P50503 | Hsc70-interacting protein |
| Q4FZU2 | Keratin, type II cytoskeletal 6A |
| Q68FS4 | Cytosol aminopeptidase |
| P47971 | Neuronal pentraxin-1 |
| P62024 | Phosphatase and actin regulator 1 |
| Q63524 | Transmembrane emp24 domain-containing protein 2 |

Supplementary Table 2 List of the unique proteins detected with at least two unique peptides in only one of the synaptosome samples

|  | **Identified only with #5 Method (n=55)** |  | **Identified only with #4 Method (n=44)** |
| --- | --- | --- | --- |
| Entry | Protein names | Entry | Protein names |
| P15429 | Beta-enolase | Q63803 | Guanine nucleotide-binding protein G(s) subunit alpha isoforms XLas |
| Q64578 | Sarcoplasmic/endoplasmic reticulum calcium ATPase 1 | P63025 | Vesicle-associated membrane protein 3 |
| Q04462 | Valine--tRNA ligase | Q66HR0 | Solute carrier family 12 member 9 |
| P14046 | Alpha-1-inhibitor 3 | P47861 | Synaptotagmin-5 |
| Q5XI83 | VPS35 endosomal protein sorting factor-like | O54861 | Sortilin |
| Q4KLP0 | Probable 2-oxoglutarate dehydrogenase E1 component DHKTD1, mitochondrial | P62919 | 60S ribosomal protein L8 |
| P26770 | Adenylate cyclase type 4 | P15178 | Aspartate--tRNA ligase, cytoplasmic |
| Q63413 | Spliceosome RNA helicase Ddx39b | Q5U2Q7 | Eukaryotic peptide chain release factor subunit 1 |
| Q63327 | Myelin-associated oligodendrocyte basic protein | Q99068 | Alpha-2-macroglobulin receptor-associated protein |
| Q6AY19 | Atypical kinase COQ8B, mitochondrial | P62282 | 40S ribosomal protein S11 |
| P40241 | CD9 antigen | P62902 | 60S ribosomal protein L31 |
| Q8R478 | WW domain-binding protein 2 | O88656 | Actin-related protein 2/3 complex subunit 1B |
| P0C0R5 | Phosphoinositide 3-kinase regulatory subunit 4 | P28042 | Single-stranded DNA-binding protein, mitochondrial |
| Q5XI31 | GPI transamidase component PIG-S | Q66H80 | Coatomer subunit delta |
| O08835 | Synaptotagmin-11 | Q6AXS5 | Plasminogen activator inhibitor 1 RNA-binding protein |
| Q6MG49 | Large proline-rich protein BAG6 | Q63450 | Calcium/calmodulin-dependent protein kinase type 1 |
| Q63357 | Unconventional myosin-Id | P84586 | RNA-binding motif protein, X chromosome retrogene-like |
| P05544 | Serine protease inhibitor A3L | Q9R080 | G-protein-signaling modulator 1 |
| Q5BJP6 | Ribosome-releasing factor 2, mitochondrial | Q6IRE4 | Tumor susceptibility gene 101 protein |
| Q6AYG5 | Ethylmalonyl-CoA decarboxylase | P60868 | 40S ribosomal protein S20 |
| Q9R085 | Ubiquitin carboxyl-terminal hydrolase 15 | Q9ESI7 | Neuronal migration protein doublecortin |
| Q5BK62 | Protein Mpv17 | P13471 | 40S ribosomal protein S14 |
| D3ZKF5 | Serine protease HTR4 | P28818 | Ras-specific guanine nucleotide-releasing factor 1 |
| Q792H5 | CUGBP Elav-like family member 2 | Q5M9G3 | Caprin-1 |
| P54777 | Peroxisome assembly factor 2 | Q9JMC1 | Phosphatidylinositol 4,5-bisphosphate 5-phosphatase A |
| B0BNM9 | Glycolipid transfer protein | Q4KM45 | UPF0687 protein C20orf27 homolog |
| Q5XHY5 | Threonine--tRNA ligase 1, cytoplasmic | P70619 | Glutathione reductase (Fragment) |
| B2RZ37 | Receptor expression-enhancing protein 5 | Q5RJK8 | Acyl-CoA-binding domain-containing protein 6 |
| Q6P7A9 | Lysosomal alpha-glucosidase | O08678 | Serine/threonine-protein kinase MARK1 |
| Q9Z0V5 | Peroxiredoxin-4 | O35889 | Afadin |
| Q6AXW1 | Glutaredoxin-2, mitochondrial | D4A631 | Brefeldin A-inhibited guanine nucleotide-exchange protein 1 |
| Q505J8 | Phenylalanine--tRNA ligase alpha subunit | Q62991 | Sec1 family domain-containing protein 1 |
| P60123 | RuvB-like 1 | P80432 | Cytochrome c oxidase subunit 7C, mitochondrial |
| Q6P7Q1 | BRISC and BRCA1-A complex member 2 | Q6NX65 | Programmed cell death protein 10 |
| P53042 | Serine/threonine-protein phosphatase 5 | Q6AYK8 | Eukaryotic translation initiation factor 3 subunit D |
| Q8K4K5 | Lethal(2) giant larvae protein homolog 1 | P63035 | Cytohesin-2 |
| F1LQ48 | Heterogeneous nuclear ribonucleoprotein L | Q66HR2 | Microtubule-associated protein RP/EB family member 1 |
| P34067 | Proteasome subunit beta type-4 | Q7TMB0 | Phospholipid phosphatase-related protein type 3 |
| Q6IMY8 | Heterogeneous nuclear ribonucleoprotein U | P62198 | 26S proteasome regulatory subunit 8 |
| Q5PPK9 | EARP and GARP complex-interacting protein 1 | Q09426 | 2-hydroxyacylsphingosine 1-beta-galactosyltransferase |
| O35412 | Signal-induced proliferation-associated 1-like protein 1 | P29534 | Vascular cell adhesion protein 1 |
| Q9QVC8 | Peptidyl-prolyl cis-trans isomerase FKBP4 | O35162 | Heat shock 70 kDa protein 13 |
| P18445 | 60S ribosomal protein L27a | Q69BT7 | Trafficking protein particle complex subunit 4 |
| P63090 | Pleiotrophin | O08697 | ADP-ribosylation factor-like protein 2 |
| Q5NDF0 | Protein O-linked-mannose beta-1,4-N-acetylglucosaminyltransferase 2 |  |  |
| P48450 | Lanosterol synthase |  |  |
| O54715 | V-type proton ATPase subunit S1 |  |  |
| Q1M168 | Caytaxin |  |  |
| Q641X8 | Eukaryotic translation initiation factor 3 subunit E |  |  |
| B5DEN9 | Vacuolar protein sorting-associated protein 28 homolog |  |  |
| P68101 | Eukaryotic translation initiation factor 2 subunit 1 |  |  |
| Q8VHV7 | Heterogeneous nuclear ribonucleoprotein H |  |  |
| O35142 | Coatomer subunit beta' |  |  |
| B5DF91 | ELAV-like protein 1 |  |  |
| Q32PX2 | Aminoacyl tRNA synthase complex-interacting multifunctional protein 2 |  |  |

|  | **Identified only with #1 Method (n=32)** | | |
| --- | --- | --- | --- |
| Entry | Protein names | Entry | Protein names |
| P22063 | Contactin-2 | P61150 | Fibroblast growth factor 12 |
| P97839 | Disks large-associated protein 4 | Q5YLM1 | Sn1-specific diacylglycerol lipase alpha |
| Q78PB6 | Nuclear distribution protein nudE-like 1 | Q9JLD2 | Neuroserpin |
| P23574 | Gamma-aminobutyric acid receptor subunit gamma-1 | Q9Z142 | Transmembrane protein 33 |
| P85972 | Vinculin | D4A6D8 | Leucine-rich repeat transmembrane neuronal protein 1 |
| Q5EB62 | Solute carrier family 25 member 46 | Q02294 | Voltage-dependent N-type calcium channel subunit alpha-1B |
| Q6MG55 | Phosphatidylserine lipase ABHD16A | Q6MG60 | N(G),N(G)-dimethylarginine dimethylaminohydrolase 2 |
| P0C0K7 | Ephrin type-B receptor 6 | P55280 | Cadherin-6 |
| Q99MI7 | NEDD8-activating enzyme E1 catalytic subunit | Q5BJR4 | Protein prune homolog 2 |
| Q63424 | Solute carrier family 15 member 2 | P26819 | Beta-adrenergic receptor kinase 2 |
| Q9WUH4 | Four and a half LIM domains protein 1 | O88881 | Brain-enriched guanylate kinase-associated protein |
| P11530 | Dystrophin | Q71RJ2 | Voltage-dependent calcium channel gamma-2 subunit |
| Q8R4E1 | T-cell immunomodulatory protein | Q8K4V4 | Sorting nexin-27 |
| B4F795 | Choline transporter-like protein 2 | P23977 | Sodium-dependent dopamine transporter |
| Q4VSI4 | Ubiquitin carboxyl-terminal hydrolase 7 | P63047 | Sulfotransferase 4A1 |
| Q66HA5 | Coiled-coil and C2 domain-containing protein 1A | Q5MPA9 | Serine/threonine-protein kinase DCLK2 |

Supplementary Table 3 Top ranking Gene Ontology terms associated with cellular compartment (SynGO).


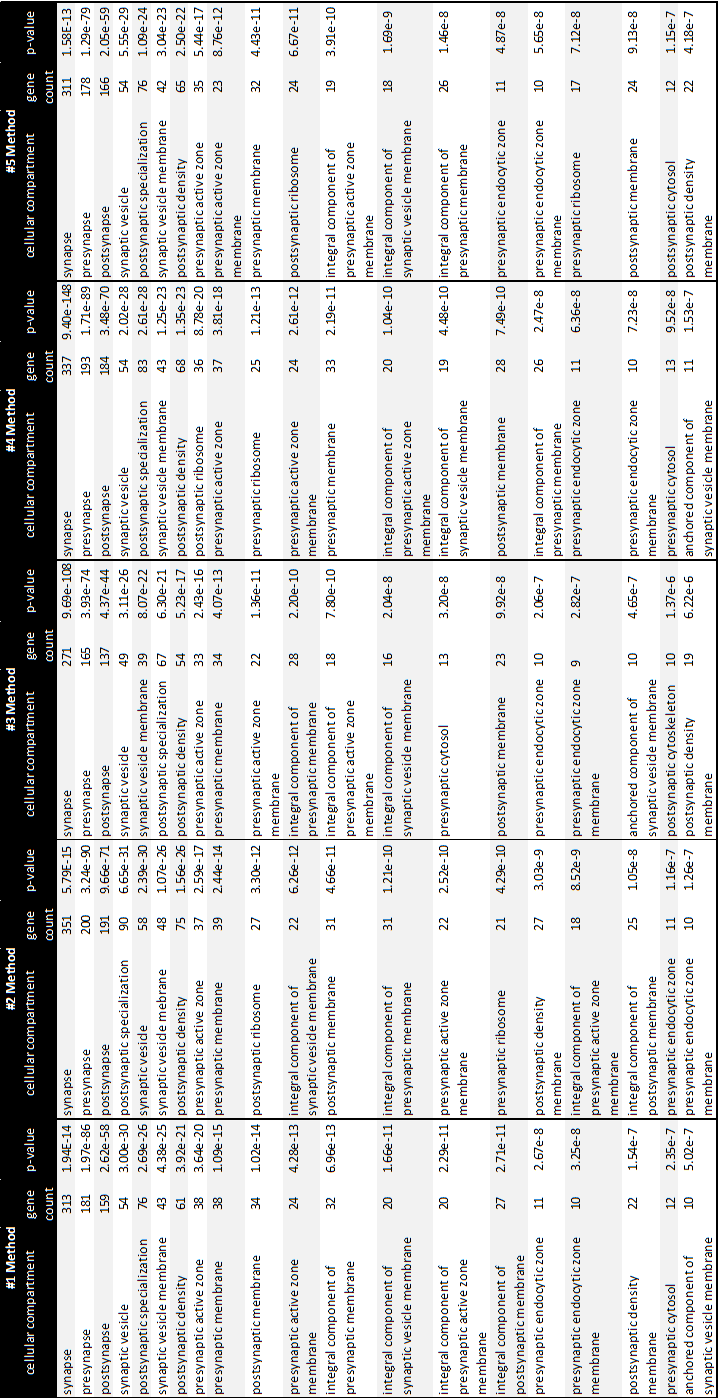


Supplementary Table 4 Top ranking Gene Ontology terms associated with biological process (SynGO).


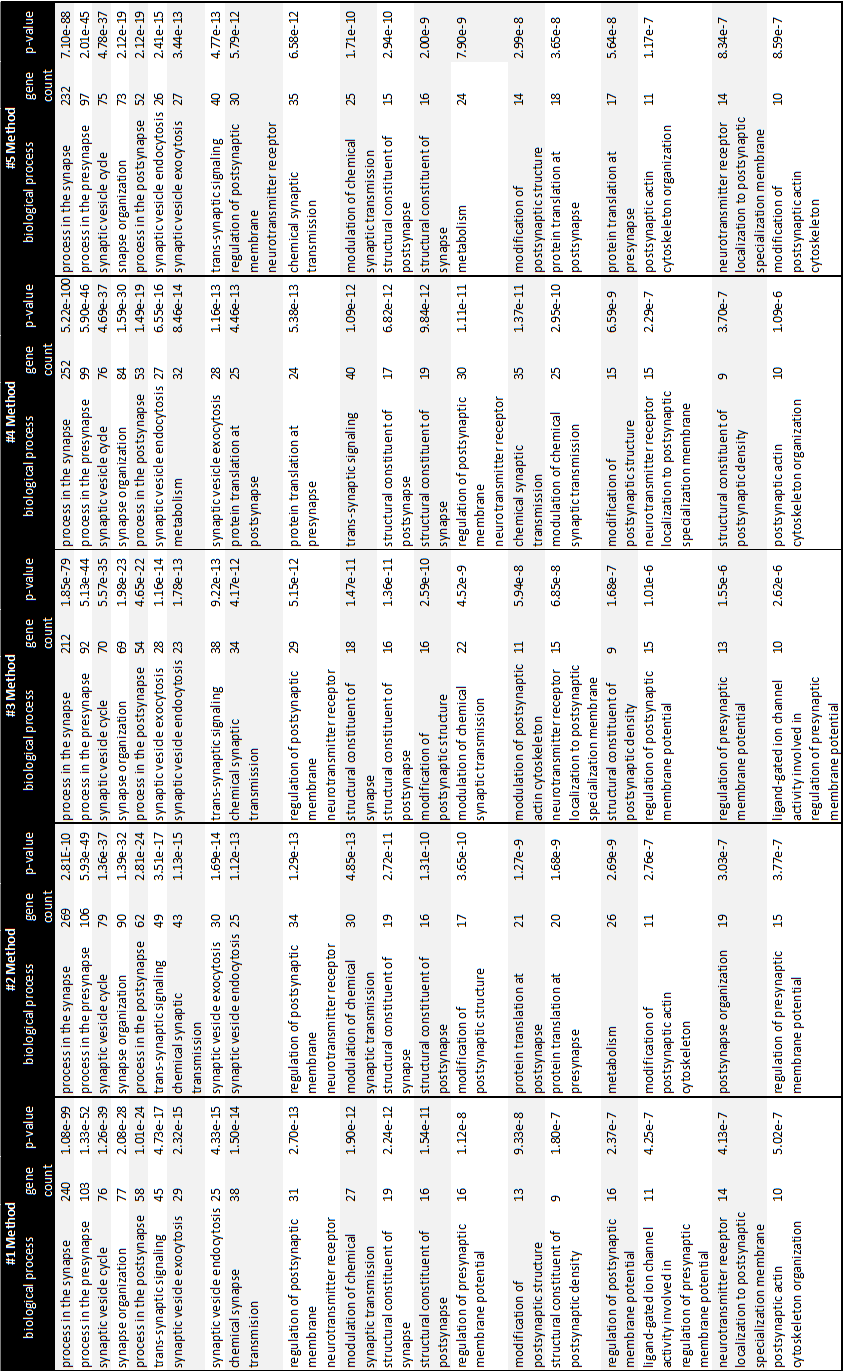


Supplementary Table 5 Top ranking Gene Ontology terms associated with cellular localization (GO:TermFinder).

Supplementary Table 6 Sub-synaptic localisation of identified proteins from the different synaptosome samples.
